# Overcoming the thermodynamic equilibrium of an isomerization reaction through oxidoreductive reactions for biotransformation

**DOI:** 10.1038/s41467-019-09288-6

**Published:** 2019-03-22

**Authors:** Jing-Jing Liu, Guo-Chang Zhang, Suryang Kwak, Eun Joong Oh, Eun Ju Yun, Kulika Chomvong, Jamie H. D. Cate, Yong-Su Jin

**Affiliations:** 1 Carl R. Woese Institute for Genomic Biology, University of Illinois at Urbana-Champaign, Urbana, IL 61801 USA; 2 Department of Food Science and Human Nutrition, University of Illinois at Urbana-Champaign, Urbana, IL 61801 USA; 3 Department of Biotechnology, Graduate School, Korea University, Seoul, 02841 South Korea; 4 National Center for Genetic Engineering and Biotechnology (BIOTEC), 113 Thailand Science Park, Phahonyothin Road, Pathum Thani, 12120 Thailand; 5 Department of Molecular and Cell Biology, University of California, Berkeley, CA 94720 USA

## Abstract

Isomerases perform biotransformations without cofactors but often cause an undesirable mixture of substrate and product due to unfavorable thermodynamic equilibria. We demonstrate the feasibility of using an engineered yeast strain harboring oxidoreductase reactions to overcome the thermodynamic limit of an isomerization reaction. Specifically, a yeast strain capable of consuming lactose intracellularly is engineered to produce tagatose from lactose through three layers of manipulations. First, *GAL1* coding for galactose kinase is deleted to eliminate galactose utilization. Second, heterologous xylose reductase (XR) and galactitol dehydrogenase (GDH) are introduced into the *∆gal1* strain. Third, the expression levels of XR and GDH are adjusted to maximize tagatose production. The resulting engineered yeast produces 37.69 g/L of tagatose from lactose with a tagatose and galactose ratio of 9:1 in the reaction broth. These results suggest that in vivo oxidoreaductase reactions can be employed to replace isomerases in vitro for biotransformation.

## Abstract (web summary)

A desired product cannot be obtained at higher concentration than its equilibrium concentration when isomerases are used for biotransformation. Here, the authors engineer in vivo oxidoreductive reactions in yeast to overcome the equilibrium limitation of in vitro isomerases-based tagatose production.

## Introduction

Thermodynamic equilibrium where concentrations of substrates and products do not change with time is a fundamental limitation of enzymatic reactions catalyzed by isomerases^1^. Biotransformations involving an isomerization reaction are therefore constrained by the underlying thermodynamic equilibrium, which often leads to an incomplete conversion of a substrate—generating a mixture of substrates and products. One typical example is the production of high-fructose corn syrup (HFCS) from glucose using glucose isomerase. Complete conversion of glucose into fructose is impossible due to the thermodynamic equilibrium of the reaction. Typically, a mixture of 42% fructose and 52% glucose is obtained by glucose isomerase^2^. In order to increase the concentration of fructose, separation and enrichment of fructose from the mixture are necessary.

Due to increasing concerns about excessive calories from added sugars in food, consumers seek a low-calorie alternative. Rare sugars such as tagatose and allulose, which possess a sweetness profile similar to sucrose but with much fewer calories, are promising low-calorie sweeteners in the food industry^3–7^. Rare sugar manufacturing usually involves isomerization reactions, resulting in a mixture of substrates and products after conversion^8–11^. As such, commercial production of the rare sugars requires additional separation steps. Manufacturing costs of rare sugars tend to be higher than HFCS and have impeded the wide use of rare sugars, even though they exhibit many benefits especially as potential anti-diabetic and anti-obesity medicines^12,13^. In particular, tagatose, a naturally occurring functional sweetener with 92% of the sweetness of sucrose but with substantially fewer calories (1.5–2.5 kcal/g vs. 4 kcal/g sucrose)^14,15^ has drawn great attention. Tagatose produced through isomerization of galactose is used as a food ingredient and attained GRAS (generally recognized as safe) status under the U.S. Food and Drug Administration (FDA). However, the manufacturing cost of tagatose by enzymatic isomerization remains high, partly due to the unfavorable thermodynamic equilibrium between galactose and tagatose^16,17^. Although the ratio of galactose and tagatose after enzymatic isomerization can be shifted to 4:6 by increasing reaction temperatures to 60 °C, the ratio of galactose and tagatose is around 7:3 under the temperatures, where L-arabinose isomerase (L-AI) is active and stable^9,18^. To obtain galactose as a substrate, enzymatic hydrolysis of lactose followed by a separation of glucose and galactose is required, and these additional steps can increase production costs. Moreover, the long-term and large-scale enzymatic conversion processes require maintenance costs, such as continued production of expensive purified enzymes. Therefore, enzyme-based tagatose production cannot compete with HFCS in the market despite its health benefits.

To bypass the above-mentioned bottlenecks, we devise a scalable and cost-effective tagatose production strategy based on direct yeast fermentation of lactose, an abundant sugar found in dairy by-products^19^. Specifically, we develope a whole-cell conversion process where lactose is transported into the cytosol of yeast, hydrolyzed, and converted into tagatose. In order to overcome the unfavorable equilibrium (7:3) by an isomerase reaction between galactose and tagatose at 30 °C, a two-step oxidoreductive pathway, consisting of aldose reductase^20^ and galactitol-2-dehydrogenase^21^, is introduced into an engineered *Saccharomyces cerevisiae* capable of assimilating lactose^19^. Additionally, to enhance the self-sustained production of tagatose in the lactose-assimilating yeast with the oxidoreductive pathway, galactose kinase (Gal1) is inactivated. As such, the resulting engineered yeast can take up lactose, produce glucose and galactose intracellularly. The intracellular glucose is used as the energy source for cell growth and maintenance, while galactose is simultaneously converted into tagatose. In this study, we demonstrate efficient tagatose production from lactose by the engineered yeast strain through a carbon-partition strategy whereby a consumed disaccharide is rerouted into different metabolic pathways—one for cell growth and maintenance, and another for targeted product generation. Interestingly, the production of tagatose directly from lactose by engineered yeast via the oxidoreductive pathway leads to a favorable conversion ratio (1:9) of galactose and tagatose, exceeding the thermodynamic equilibrium (7:3) by an isomerase reaction at 30 °C.

## Results

### A metabolic design to accumulate intracellular galactose

**Fig. 1:**
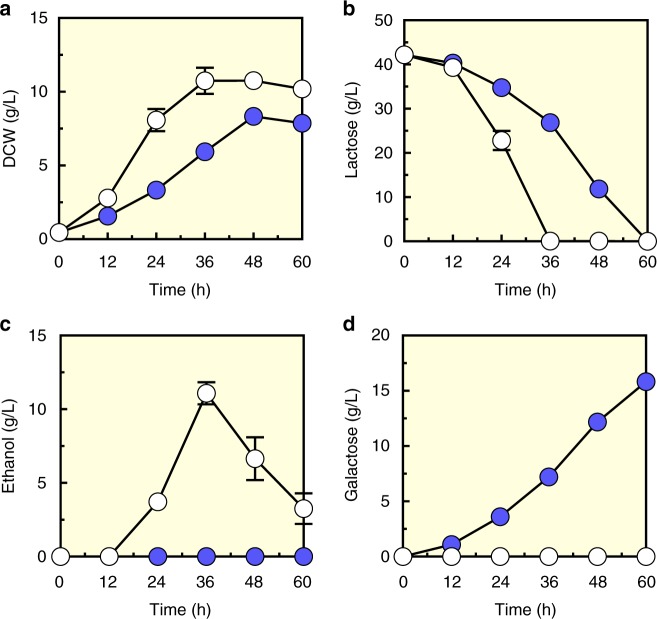
Galactose accumulation in lactose-consuming strain EJ2 with *GAL1* deletion (EJ2g). **a** Growth profile as shown by dry cell weight (DCW), **b** lactose consumption, **c** ethanol production, and **d** galactose production by the EJ2g strain (filled circles) as compared with the EJ2 control stain (open circles) under aerobic conditions. Data are presented as mean value and standard deviations of three independent biological replicates. Source data are provided as a Source Data file

Previously, we constructed an engineered yeast strain (EJ2) to utilize cellobiose by introducing cellobiose transporter (CDT-1) and intracellular β-glucosidase (GH1-1)^22^. Notably, we found that the EJ2 strain also used lactose intracellularly^19^, suggesting that CDT-1 can import lactose and GH1-1 can hydrolyze lactose as well as cellobiose. To accumulate intracellular galactose, we deleted the gene *GAL1* coding for galactose kinase, which catalyzes the first step of the Leloir pathway responsible for galactose assimilation in yeast^23^. As expected, the *GAL1*-deleted strain (EJ2g) grew well on lactose, but accumulated galactose during lactose utilization (Fig. 1 and Supplementary Figure 1) in comparison with the parental strain (EJ2). No glucose accumulated in the culture medium with either strain. This result indicated that after lactose was hydrolyzed into glucose and galactose intracellularly, glucose supported cell growth of the EJ2g strain while galactose was not further metabolized. We therefore used the EJ2g strain as a host strain for intracellular conversion of galactose into tagatose.

### Conversion of galactose into tagatose by oxidoreductases

**Fig. 2:**
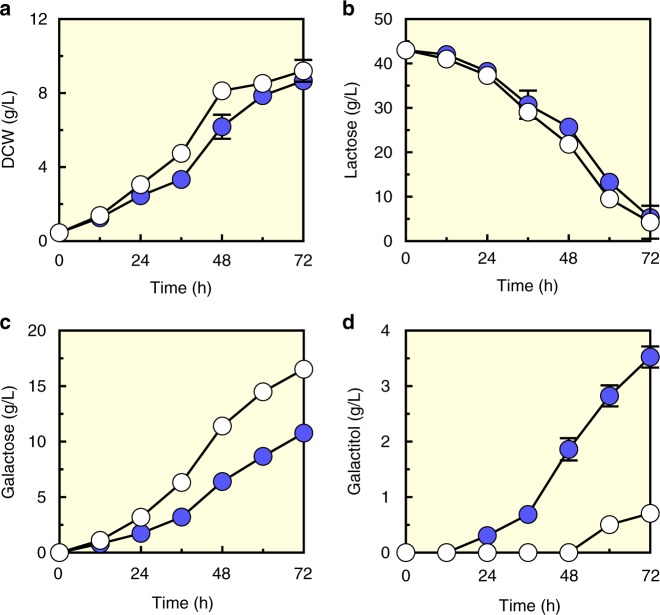
Galactitol production after xylose reductase (XR) introduction into EJ2g. **a** Growth profile as shown by dry cell weight (DCW), **b** lactose consumption, **c** galactose production, and **d** galactitol produciton by the EJ2g strain with XR overexpression in the pRS42K plasmid (EJ2g_pX, filled circles), and EJ2g with empty plasmid pRS42K as control (open circles) on YP medium with 40 g/L lactose under aerobic conditions. Data presented as mean values and standard deviations of three independent biological replicates. Source data are provided as a Source Data file

**Fig. 3:**
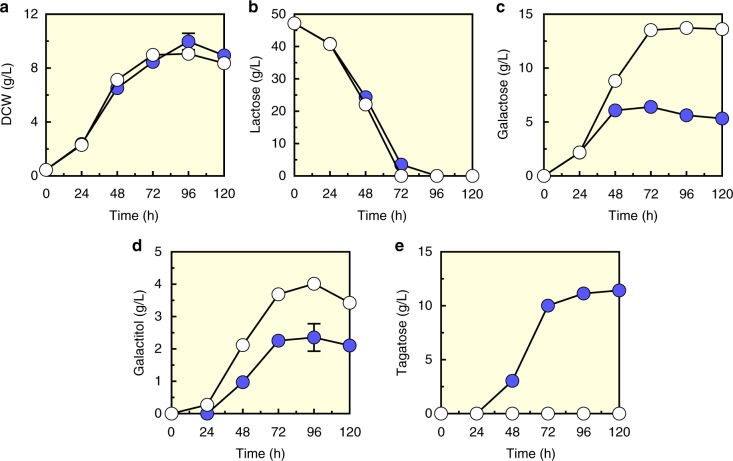
Tagatose production after the introduction of galactitol-2-dehydrogenase (GDH). **a** Growth curve as shown by DCW, **b** lactose consumption, **c** galactose production, and **d** galactitol production, and **e** tagatose production by the EJ2g_pXpG strain (filled circles) and the EJ2g_pX strain with p42H empty plasmid as control (open circles) under aerobic condition. Data are presented as mean value and standard deviations of three independent biological replicates. Source data are provided as a Source Data file

To overcome the unfavorable equilibrium between galactose and tagatose by an isomerization reaction, we employed two oxidoreductases commonly used for the isomerization of aldose and ketose in yeast and fungi for the intracellular conversion of galactose into tagatose. As xylose reductase (XR) is known to reduce galactose into galactitol^20^, *Scheffersomyces stipitis XYL1* coding for NADPH-linked XR was expressed in the EJ2g strain to produce galactitol from lactose. Expressing XR using a multicopy plasmid (pRS42K) and a strong constitutive promoter (P_*TDH3*_) (EJ2g_pX) resulted in the production of 3.5 g/L of galactitol during lactose fermentation (Fig. 2 and Supplementary Figure 2). To convert galactitol into tagatose, an NAD^+^-linked galactitol-2-dehydrogenase (GDH) from *Rhizobium leguminosarum*^21^ was expressed in the galactitol-producing strain (EJ2g_pX) using a strong promoter (P_*TDH3*_) and a multicopy plasmid (pRS42H), resulting in the EJ2g_pXpG strain. The EJ2g_pXpG strain produced 11.43 g/L of tagatose from 47.2 g/L of lactose (Fig. 3 and Supplementary Figure 4). These data demonstrated that the two oxidoreductases (XR and GDH) functionally expressed in the engineered yeast (EJ2g_pXpG) and they enabled direct production of tagatose from lactose.

### Fine-tuning of XR and GDH for enhanced tagatose production

**Fig. 4:**
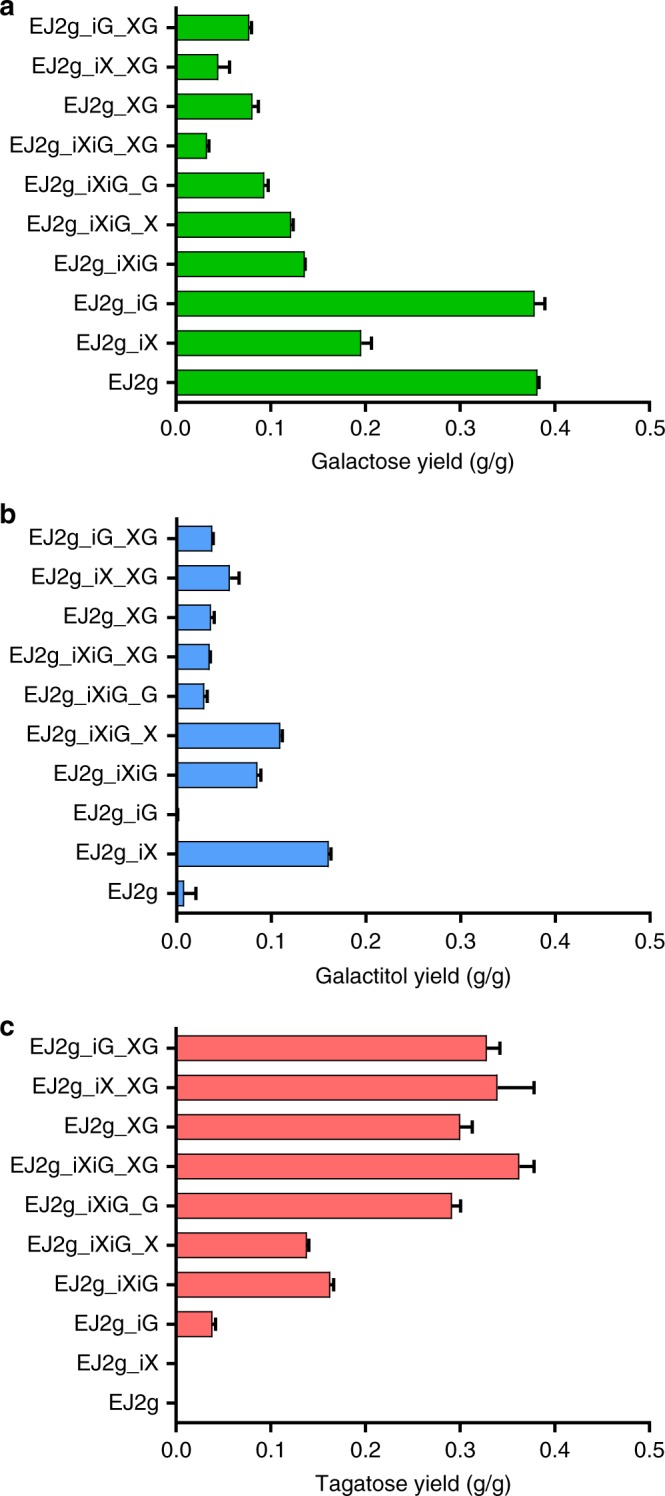
Improved production of tagatose by increasing the XR and GDH gene copy numbers. **a** Galactose yield, **b** galactitol yield, and **c** tagatose yield. Data are presented as mean value and standard deviations of three independent biological replicates. Source data are provided as a Source Data file

While the engineered yeast EJ2g_pXpG produced tagatose from lactose, the observed yield (0.242 g tagatose/g lactose) was only half of the theoretical yield (0.526 g tagatose/g lactose). We reasoned that the XR and GDH expression levels might need optimization to minimize the formation of by-products, such as galactose and galactitol (Fig. 3b, c), and maximize tagatose production. To elucidate the relationships between the galactose, galactitol, and tagatose production levels and the expression levels of XR and GDH, we constructed three more engineered strains with additional copies of XR and GDH via Cas9-based genome integration^24,25^. The production of galactose, galactitol, and tagatose from lactose by the four engineered yeast strains EJ2g_pXpG, EJ2g_iXiG_pX, EJ2g_iXiG_pG, and EJ2g_iXiG_pXpG (Table 1) was measured by HPLC (Fig. 4 and Supplementary Figure 5). Additional overexpression of XR in the EJ2g_iXiG_pX strain resulted in increased galactitol yield, but the yield of tagatose was unchanged. By contrast, additional overexpression of GDH in the EJ2g_iXiG_pG strain led to less galactitol accumulation and more tagatose production than the parental strain EJ2g_iXiG. The EJ2g_iXiG_pXpG strain with overexpression of both XR and GDH exhibited the highest tagatose and lowest galactitol yields (Fig. 4). These results suggested that XR and GDH expression levels played a critical role in manipulating the metabolic fluxes toward tagatose production. Using simple modifications in the expression levels of XR and GDH, we increased the yield of tagatose from lactose by more than 20%. The final galactose to tagatose ratio of EJ2g_iXiG_pXpG strain in the fermentation broth was 2:8.

**Table 1:** Engineered *S. cerevisiae* strains used in this study

Strains	Description	Source
EJ2	Evolved strain of EJ1 (D452-2 leu2:LEU2 pRS405-gh1-1 ura3:URA3 pRS406-cdt-1)	^22^
EJ2g	EJ2 Δgal1	This study
EJ2g_pX	EJ2g w/p42K-XR	This study
EJ2g_pXpG	EJ2g w/p42K-XR and p42H-GDH	This study
EJ2g_iX	EJ2g w/integrated pTDH3-XYL1-tTDH3 in the CS6 locus	This study
EJ2g_iG	EJ2g w/integrated pTDH3-GDH-tCYC1 in the CS8 locus	This study
EJ2g_iXiG	EJ2g w/integrated pTDH3-XYL1-tTDH3 and pTDH3-GDH-tCYC1	This study
EJ2g_iXiG_pX	EJ2g_iXiG w/p42K-XR	This study
EJ2g_iXiG_pG	EJ2g_iXiG w/p42H-GDH	This study
EJ2g_iXiG_pXpG	EJ2g_iXiG w/p42K-XR and p42H-GDH	This study
EJ2g_iX_pXpG	EJ2g_iX w/p42K-XR and p42H-GDH	This study
EJ2g_iG_pXpG	EJ2g_iG w/p42K-XR and p42H-GDH	This study

### Tagatose production in a bioreactor

**Fig. 5:**
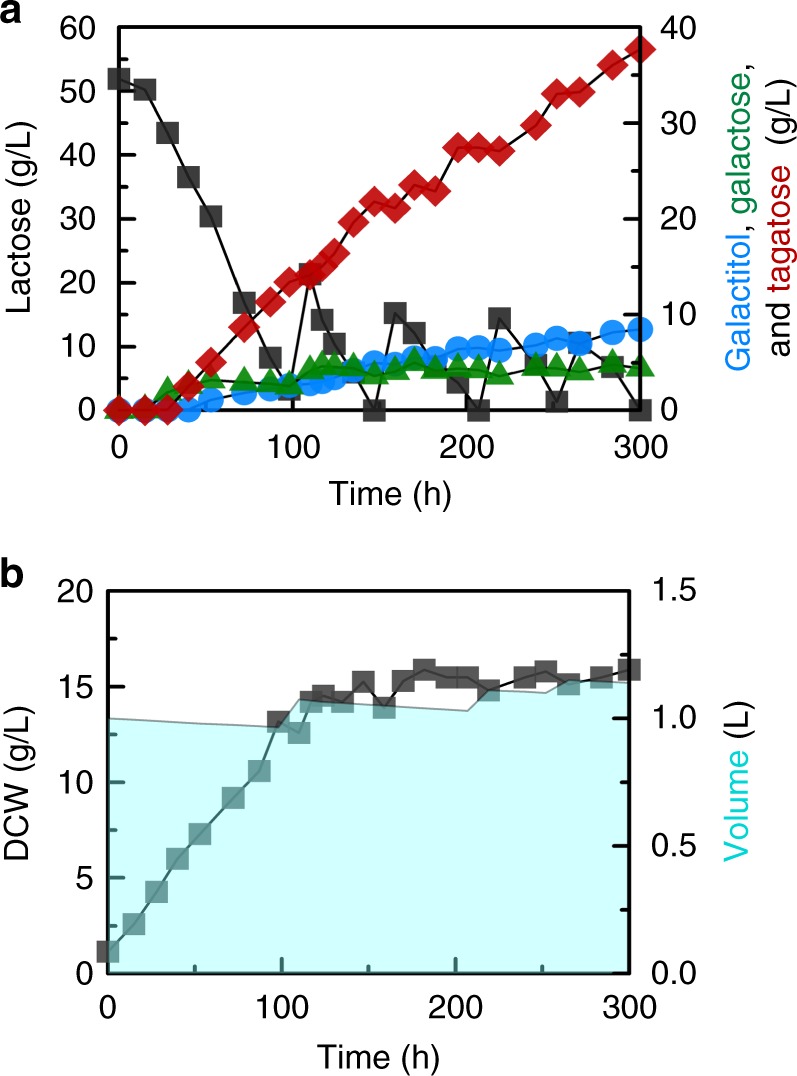
Tagatose production by the EJ2g_iXiG_pXpG strain in a 2 L bioreactor. **a** Profiles of lactose, galactose, galactitol, and tagatose concentrations, and **b** the growth of the EJ2g_iXiG_pXpG strain and the volume changes of bioreactor during the fed-batch fermentation. Source data are provided as a Source Data file

To increase the titer of tagatose and to investigate the feasibility of large-scale production of tagatose from lactose, the EJ2g_iXiG_pXpG strain exhibiting the highest tagatose titer from a shake flask fermentation was tested in a bioreactor (Fig. 5 and Supplementary Figure 6). After 50 g/L of lactose was consumed, additional feedings of lactose were conducted to maintain lactose concentrations around 10–20 g/L. After the fed-batch fermentation, 114.21 g/L of lactose was consumed and the titers of tagatose, galactose, and galactitol were 37.69 g/L, 4.41 g/L, and 8.46 g/L, respectively. As such, the conversion ratios of tagatose, galactose, and galactitol were 74.5%, 8.7%, and 16.7%, respectively. The tagatose yield from lactose was 0.33 g tagatose/g lactose, which is equivalent to 62.7% of a theoretical maximum (0.526 g tagatose/g lactose). Furthermore, the ratio of galactose to tagatose in the fermentation broth reached as high as 1:9 which is much higher than the ratio of 2:8 from the shake flask fermentation, substantially improving the 7:3 ratio achieved with the isomerase pathway at 30 °C. The concentration of galactose reached a steady-state level during the lactose feeding process (Fig. 5), suggesting that galactose can be converted efficiently into galactitol and tagatose with the lactose feeding rates used in this experiment. The galactitol yield in the fed-batch fermentation (0.074 g galactitol/g lactose vs. 0.035 g galactitol/g lactose) was higher than that in the shake flask fermentation, suggesting that GDH activity and supply of NAD^+^ might need to be further optimized to cope with higher metabolic fluxes from lactose and galactose.

## Discussion

**Fig. 6:**
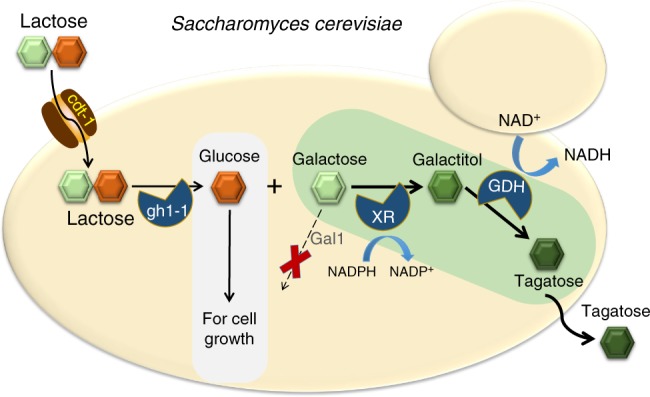
Schematic diagram of the production of tagatose from lactose in engineered *S. cerevisiae*. Heterologous expression of cdt-1 (cellobiose transporter), gh1-1 (β-glucosidase), XR (xylose reductase), GDH (galactitol-2-dehydrogeanse), and deletion of Gal1 (galactose kinase) were combined in *S. cerevisiae* to produce tagatose directly from lactose

In this study, we bypassed the thermodynamic equilibrium of the isomerase reaction by using an oxidoreductive pathway to achieve the one-pot biosynthesis (Fig. 6). Tagatose production was selected as a demonstration system as its production was shown previously to be limited by the thermodynamic equilibrium^8,9,18,26–28^.

Interconversion between aldose and ketose forms of sugars are critical for their assimilation by microorganisms. Both single-step isomerization and two-step oxidoreductive reactions have evolved to catalyze these interconversions. For instance, xylose can be converted into xylulose via either xylose isomerase (XI) or the combined action of xylose reductase (XR) and xylitol dehydrogenase (XDH)^29^. The XI reaction is often associated with anaerobic or microaerophilic bacteria^30,31^, due to the fact that the isomerase enzyme does not require cofactors and can carry out bioconversions under oxygen-limited and anaerobic conditions. Isomerase reactions have been adopted for industrial production of various value-added chemicals, but face two fundamental drawbacks in their use. First, the inherent thermodynamic equilibrium between the substrate and product leads to low conversion yields and creates difficulties for downstream products separation and purification. Second, the scale-up cost sharply increases, because the amount of enzyme required is directly proportional to the proposed reaction scale. For example, rare sugars such as tagatose and allulose are currently produced by enzymatic reactions followed by complicated separation processes. As a result, the production costs of rare sugars are significantly higher than HFCS, which does not require additional separation. Due to this high production cost, introduction of rare sugars into foods and beverages has been hampered in spite of potential benefits of rare sugars^4^.

Unlike the isomerase reaction, the oxidoreductive reaction consists of two steps (an oxidation and a reduction) and requires cofactors. Though seemingly redundant, this mechanism prevents the undesired reverse reaction through thermodynamic coupling to cofactor equilibria. Therefore, we used the oxidoreductive pathway instead of the isomerase pathway, to achieve more complete conversion of galactose into tagatose product than the isomerization. To implement the oxidoreductive pathway for tagatose production, in vivo bioconversion is more suitable than in vitro enzymatic conversion, considering the need for redox cofactors. Simultaneously, an efficient self-sustained bioconversion system is advantageous when considering scale-up costs. While direct and efficient conversion of lactose into tagatose by the  oxidoreductive pathway has been demonstrated in this study, the secretion of galactose and galactitol during the conversion needs to be addressed in order to reach the theoretical maximum yield. We speculate that the secretion of galactose and galactitol might be facilitated by endogenous hexose and sugar alcohol transporter in yeast. Specifically, Gal2 (galactose permease) might be responsible for the secretion and re-assimilation of galactose, and Fps1 (aquaglyceroporin), which have been reported to transport xylitol^32^, might be involved in the secretion of galactitol. From the structural similarity between galactitol and xylitol, the deletion of *FPS1* in a tagatose-producing strain might reduce production of galactitol. As Gal2 might be involved in both secretion and re-assimulation of galactose, upregulation of *GAL2* might increase the secretion and re-assimilation of galactose, and deletion might reduce the secretion and re-assimilation of galactose. As such, the effects of *GAL2* perturbation would likely be mixed or compromised. Genetic perturbations of endogenous sugar and sugar alcohol transporters including *FPS1* and *GAL2* can be conducted in the future to further improve the conversion yield of tagatose from lactose. In addition to galactitol accumulation as a by-product, the productivity and titer of tagatose from lactose by our engineered yeast need to be further improved. While the productivity (0.126 g/L·h) and titer (37.69 g/L) of tagatose production from lactose by our engineered yeast are comparable with the reported productivities (0.103–0.896 g/L·h) and titers (14.8–21.5 g/L) by enzyme-based tagatose production from lactose^18,33^, they are much lower than the productivity (7.9 g/L·h) and titer (158 g/L) by enzyme-based tagatose production from galactose^5^. Continuous fermentation with cell-recycling might be a possible approach to improve the productivity of tagataose production from lactose by our engineered yeast.

We also designed a carbon partition strategy that utilizes lactose, a disaccharide of galactose, and glucose, as an initial substrate for simultaneous tagatose production and glucose catabolism. Specifically, we intentionally shut down the native galactose utilization pathway of *S. cerevisiae* and redirected galactose toward tagatose through the introduced oxidoreductive pathway. Meanwhile, glucose was consumed to sustain the engineered yeast via its native pathway. Because the two monosaccharides were released intracellularly, the glucose repression on galactose uptake was bypassed and thus allowed for simultaneous utilization^34,35^. Hydrolysis of lactose into glucose and galactose, and the subsequent conversion of galactose to tagatose, were integrated and self-sustained, which dramatically reduced processing costs. It is worth noting that the carbon-partition strategy is not only limited to tagatose production but can also be applied in other bioconversion systems. When consuming disaccharides, we can opt to turn off the native catabolic pathway of one of the monosaccharide moieties and reprogram this monosaccharide toward our target chemicals, while leaving another moiety for cell growth and maintenance. If it is simplier to produce the target chemical using glucose rather than galactose as a substrate, we can turn off the glucose pathway by disrupting the hexose kinases (encoded by *HXK1* and *HXK2*) and glucose kinase (encoded by *GLK1*). We can then introduce a target oxidoreductive pathway to allow glucose rerouting to the target chemicals, while maintaining the native galactose pathway for cell maintenance. Apart from lactose, other disaccharides such as sucrose are also cheap and abundant. Many other engineered yeast strains rapidly consume these disaccharides as well. Thus, we can produce target chemicals from these disaccharides as needed through the carbon-partition strategy.

In this study, our strategy reduces the production cost at every step, as compared with existing industrial tagatose production systems. First, the traditional process requires that the majority of galactose is made from enzymatic hydrolysis of lactose, followed by the separation of glucose and galactose. Therefore, direct consumption of lactose by our engineered yeast strain can significantly reduce the enzyme (β-galactosidase) and separation costs. In addition, lactose is readily obtained from whey, an abundant waste product from cheese and Greek yogurt production. Next, the in vivo oxidoreductive conversion of galactose into tagatose eliminated the cost of purified or immobilized L-arabinose isomerase. Unlike the traditional process, where enzyme cost increases proportionally with the scale of operation, the engineered yeast replicates itself continuously regardless of the reaction scale. Most importantly, the oxidoreductive reaction allows near-complete conversion of galactose into tagatose. The small amounts of galactose in the fermentation broth can be completely removed by adding regular yeast that consumes galactose to simplify the downstream separation process. Therefore, our strategy not only maximizes substrate value, but also simplifies product separation and purification, resulting in an overall decrease in production costs. In future studies, we intend to optimize the oxidoreductive galactose–tagatose pathway, to increase tagatose yields, to achieve complete conversion of galactose into tagatose, and to further minimize the separation and purification cost. In summary, we envision the carbon-partition strategy applicable for value-added chemical productions from disaccharides by engineered microorganisms. Our scheme allows for the disaccharides to be utilized intracellularly, and its monosaccharide products allotted for maintenance and production separately, but simultaneously.

## Methods

### Strains and media

*Escherichia coli* Top10 (Invitrogen, Grand Island, NY) was used for the construction and propagation of plasmids. *E. coli* was grown in the Luria-Bertani medium (5 g/L yeast extract, 10 g/L tryptone, 10 g/L NaCl, pH 7.0) at 37 °C, and ampicillin (100 μg/mL) was added for selection when required. Yeast strains were grown on the YPD medium (10 g/L yeast extract, 20 g/L peptone, and 20 g/L glucose) at 30 °C. Yeast strains transformed with plasmids containing antibiotic markers were propagated on YPD plates supplemented with the corresponding antibiotics, such as hygromycin B (300 μg/mL) and/or geneticin G418 (300 μg/mL).

### Plasmids and strain construction

The guide RNA plasmid p42H-gGAL1 (Supplementary Table 3) was amplified from gRNA-ura-HYB^25,36^ used as a template by using primer pair Gal1-gU and Gal1-gD (Supplementary Table 1) carrying a 20 bp PAM sequence for *GAL1* deletion. Donor DNA was amplified using primers Gal1-Donor-U and Gal1-Donor-D (Supplementary Table 1). *GAL1* was deleted in the EJ2 strain^22^ using CRISPR–Cas9 technology^25^. Plasmid CAS9-NAT (Supplementary Table 3) was transformed into the EJ2 strain followed by guide RNA plasmid (p42H-gGAL1) and donor DNA transformation. The transformants were diagnosed for *GAL1* deletion using primers Gal1-CK-U and Gal1-CK-D (Supplementary Table 1). Plasmid pYS10^37^ was digested by *Sac*I and *Xho*I, and the pTDH3-XYL1-tTDH3 cassette was ligated with the same enzyme-digested pRS42K to construct p42K-XR (Supplementary Table 3). The pGPD-tCYC1 cassette from plasmid p426-pGPD^38^ was double digested by *Sac*I and *Kpn*I and ligated with *Sac*I and *Kpn*I digested pRS42H^39^, forming plasmid p42H-pGPD (Supplementary Table 3). The gene fragment of GDH was synthesized as gBlocks (IDT Inc, Skokie, IL) and blunt-ligated with plasmid p42H-pGPD to generate plasmid p42H-GDH. In order to integrate target genes into the genome of strain EJ2g for stable expression, CS6 and CS8 loci were used. The CS6 and CS8 sites are selected intergenic regions for integration of expression cassettes via Cas9-based genome editing. The CS6 site is located between *YGR190C* and *tW(CCA)G2* in Chromosome VII, and the CS8 is located between *YPR014C* and *YPR015C* in Chromosome XVI. Targeting guide RNA sequences for CS6 and CS8 are listed in Supplementary Table 2. Detailed sequence information of the CS6 and CS8 sites are provided in Supplementary Data 1 and Supplementary Data 2. Based on the targeting sequences, guide RNA-expressing plasmids (p42K-CS8 and p42K-CS6) were constructed by reverse PCR of a pRS42K plasmid-containing guide RNA sequence using primer pairs gCS6-U/gCS6-D and gCS8-U/gCS8-D, respectively (Supplementary Table 1)^40^.

For genomic integration of *XYL1* into the EJ2g strain, the plasmid p42K-XR was amplified using a primer pair of CS6-IU and CS6-ID as donor DNAs for CRISPR–Cas9-based integration. The transformants with CS6-XR integration were confirmed by PCR using primers CS6-CKU and CS6-CKD, and was designated as the EJ2g_iX strain (Table 1). Similarly, CS8-GDH was integrated into the EJ2g_iX strains with a donor DNA amplified from plasmid p42H-GDH using primers CS8-IU and CS8-ID. The transformants were confirmed by PCR using primers CS8-CKU and CS8-CKD, and the engineered strain with CS6-XR and CS8-GDH integration was named as the EJ2g_iXiG strain (Table 1).

### Yeast culture and fermentation conditions

The lactose fermentation was started by inoculating the overnight yeast pre-culture (5 mL of the YP medium containing 20 g/L of cellobiose) into 20 mL YPL (the YP medium containing 40–50 g/L of lactose) in a 125 mL Erlenmeyer flask to obtain an initial optical density at 600 nm (OD_600_) = 1.0 then incubated at 30 °C with shaking (orbit of 2.5 cm). The engineered yeast strain was precultured in 100 mL of the YP medium containing 20 g/L of lactose with hygromycin (300 μg/mL) and G418 (300 μg/mL) at 30 °C with shaking (orbit of 2.5 cm) with baffled flasks for 2 days. Cells at stationary phase were harvested at 15,294×*g* for 2 min, and suspended in sterilized water before inoculation. Fed-batch culture was conducted in BioFlo & CelliGen (R) 310 fermenter (New Brunswick Scientific-Eppendorf) with the YP medium containing 50 g/L of lactose with 300 μg/mL of hygromycin and 300 μg/mL of G418 at 30 °C. The initial cell density was OD_600_ = 2.5. YP medium with 200 g/L of lactose containing antibiotics was slowly fed through a pump to maintain the lactose concentration at 10–20 g/L. The feeding rate was manually adjusted based on lactose consumption and lactose concentration in the fermenter. The culture pH was automatically maintained at 5.6 by adding 3 N NaOH or HCl solution. The gas flow rate was set at 1 vvm, and agitation was set at 800 rpm.

### Analytical methods and metabolite analysis

The OD_600_ of cultures was measured using a spectrophotometer (Biomate 5, Thermo, NY) and dried cell weight (DCW) was obtained from an experimentally determined conversion factor of 0.454 g DCW/OD_600_. Extracellular metabolites such as lactose, galactose, galactitol, glycerol, acetate, and ethanol were measured by HPLC (Agilent Technologies 1200 Series, Santa Clara, CA) with a Rezex^TM^ ROA-Organic Acid H + (8%) column (Phenomenex Inc., Torrance, CA) and a refractive index detector (RID). The column was eluted with 0.005 N H_2_SO_4_ at a flow rate of 0.6 mL/min at 50 °C. Galactose and tagatose could not be separated by this ROA-organic acid H + (8%) column (Supplementary Figure 3). Galactose and tagatose concentrations were measured using an Agilent Technologies 1200 Series HPLC equipped with a Rezex^TM^ RCM-Monosaccharide Ca + 2 (8%) column (Phenomenex Inc. Torrance, CA) and RID detector. The mobile phase was E-Pure™ Water (Barnstead E-pure™ Water Purification Systems, Thermo Scientific) and was eluted at a flow rate of 0.6 mL/min at 80 °C. The maxium theoretical tagatose yield (0.526 g tagatose/g lactose) by engineered yeast was calculated based on the molecular weights of tagatose (180.16 g/mol) and lactose (342.3 g/mol) with an assumption that glucose is not converted into tagatose.

## Supplementary information

Suplementary Information

Peer Review File

Description of Additional Supplementary Files

Supplementary Data 1

Supplementary Data 2

Source Data

## Data Availability

Data supporting the findings of this work are available within the paper and its Supplementary Information files. A reporting summary for this article is available as a Supplementary Information file. The source data underlying Figs. 1, 2, 3, 4, and 5 are provided as a Source Data file. All other data are available from the corresponding author upon reasonable request.
