# Supplementary material for: Overcoming the thermodynamic equilibrium of an isomerization reaction through oxidoreductive reactions for biotransformation
Source: Nat Commun. 2019 Mar 22;10:1356. doi: 10.1038/s41467-019-09288-6 (PMC6430769; doi:10.1038/s41467-019-09288-6)
Supplement: Supplementary file 1 — Suplementary Information [file 41467_2019_9288_MOESM1_ESM.pdf]

Supplementary Informaotion

Overcoming the thermodynamic equilibrium of an isomerization reaction through oxidoreductive reactions for biotransformation

Jing-Jing Liu [a], Guo-Chang Zhang [a,b], Suryang Kwak [a,b], Eun Joong Oh [a,b], Eun Ju Yun [a,c], Kulika Chomvong [d], Jamie H. D. Cate [e], Yong-Su Jin [a,b,*]

[a] Carl R. Woese Institute for Genomic Biology, University of Illinois at Urbana-Champaign, Urbana, IL, 61801, USA

[b] Department of Food Science and Human Nutrition, University of Illinois at Urbana-Champaign, Urbana, IL, 61801, USA

[c] Department of Biotechnology, Graduate School, Korea University, Seoul, 02841, South Korea

[d] National Center for Genetic Engineering and Biotechnology (BIOTEC), 113 Thailand Science Park, Phahonyothin Road, Pathum Thani 12120, Thailand

[e] Department of Molecular and Cell Biology, University of California, Berkeley, CA, 94720, USA

* Corresponding author: Yong-Su Jin

Tel: 217-333-7981, Fax: 217-333-0508, Email: ysjin@illinois.edu.

Mailing address: 1206 W. Gregory Drive, Carl R. Woese Institute for Genomic Biology, Urbana, IL 61801, United States

Supplementary Table 1 Primers used in this study.

Primers	Primer sequences	Source
Gal1-gU	aaaggaattaccaagaccatgttttagagctagaaatagcaag	This study
Gal1-gD	atggtcttggtaattcctttgatcatttatctttcactgcgga	This study
Gal1-Donor-U	gtatcaacaaaaaattgttaatatacctctatactttaacgtcaaggagaaaaaactatag tatacttctttttt	This study
Gal1-Donor-D	aagttatgagtagaaaaaaatgagaagttgttctgaacaaagtaaaaaaaagaagtata ctatagtttttctcc	This study
Gal1-CK-U	ctgaaacgcagatgtgcctcg	This study
Gal1-CK-D	ggtagtcatatcatgtcaag	This study
gCS8-U	TGATTCAATCATTCTTATTGgttttagagctagaaatagcaag	[1,2]
gCS8-D	CAATAAGAATGATTGAATCAgatcatttatctttcactgcgga	[1,2]
CS8-IU	caaaattacctacggtaattagtgaaaggccaaaatctaatgttacaataAATTAA CCCTCACTAAAGGGA	[1,2]
CS8-ID	gaccgttcccttgtgttgtaccagtggtagggttcttctcggtagcttctGTAATAC GACTCACTATAGGGC	[1,2]
CS8-CKU	agtggaacatagaagggg	[1,2]
CS8-CKD	Taagcagcccagtgaac	[1,2]
gCS6-U	GATACTTATCATTAAGAAAAgttttagagctagaaatagcaag	[2]
gCS6-D	TTTTCTTAATGATAAGTATCgatcatttatctttcactgcgga	[2]
CS6-IU	aacctcgaggagaagttttttaccccctctccacagatcCAGGAAACAGCT ATGACCATG	[2]
CS6-ID	taattaggtagaccgggtagatttttccgtaaccttggtgtcTGTAAAACGAC GGCCAGT	[2]
CS6-CKU	gtctgccgaaattctgtg	[2]
CS6-CKD	cggtcagaaagggaaatg	[2]

Supplementary Table 2 The targeting guide RNA sequences used in this study.

Intergenic site	gRNA target sequence
CS6	GATACTTATCATTAAGAAAA
CS8	TGATTCAATCATTCTTATTG

Supplementary Table 3 Plasmids used in this study.

Plasmids	Description	Source
pRS42K	pRS42K	[3]
pRS42H	pRS42H	[3]
pYS10	pRS305 pTDH3-XYL1-tTDH3	[4]
p42K-XR	pRS42K pTDH3-XYL1-tTDH3	This study
p426-pGPD	pSR426 pTDH3-tCYC1	[5]
p42K-pGPD	pRS42K pTDH3-tCYC1	[1]
p42H-pGPD	pRS42H pTDH3-tCYC1	This study
p42H-GDH	pRS42H pTDH3-GDH-tCYC1	This study
CAS9-NAT	p414-TEF1p-Cas9-CYC1t-NAT1	[1]
p42H-gGAL1	pRS42H carrying guide RNA for integration at CS6 locus	This study
p42K-gCS8	pRS42K carrying guide RNA for integration at CS8 locus	[1,2]
p42H-gCS6	pRS42H carrying guide RNA for integration at CS6 locus	[2]

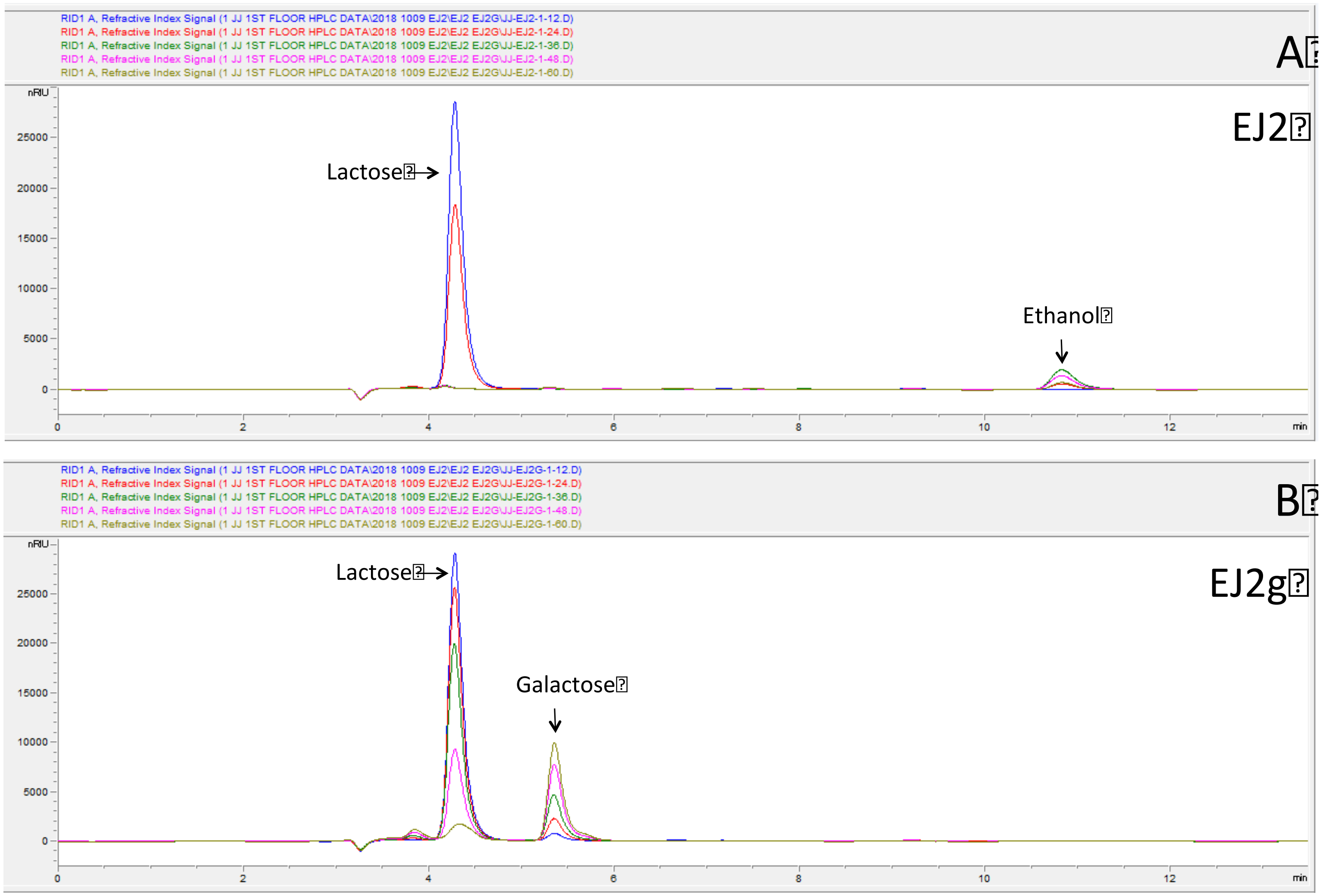

Supplementary Figure 1 HPLC chromatograms of the fermentation samples (A: EJ2, B: EJ2g) for Fig. 1 (one representative figure of triplicate samples during fermentation).

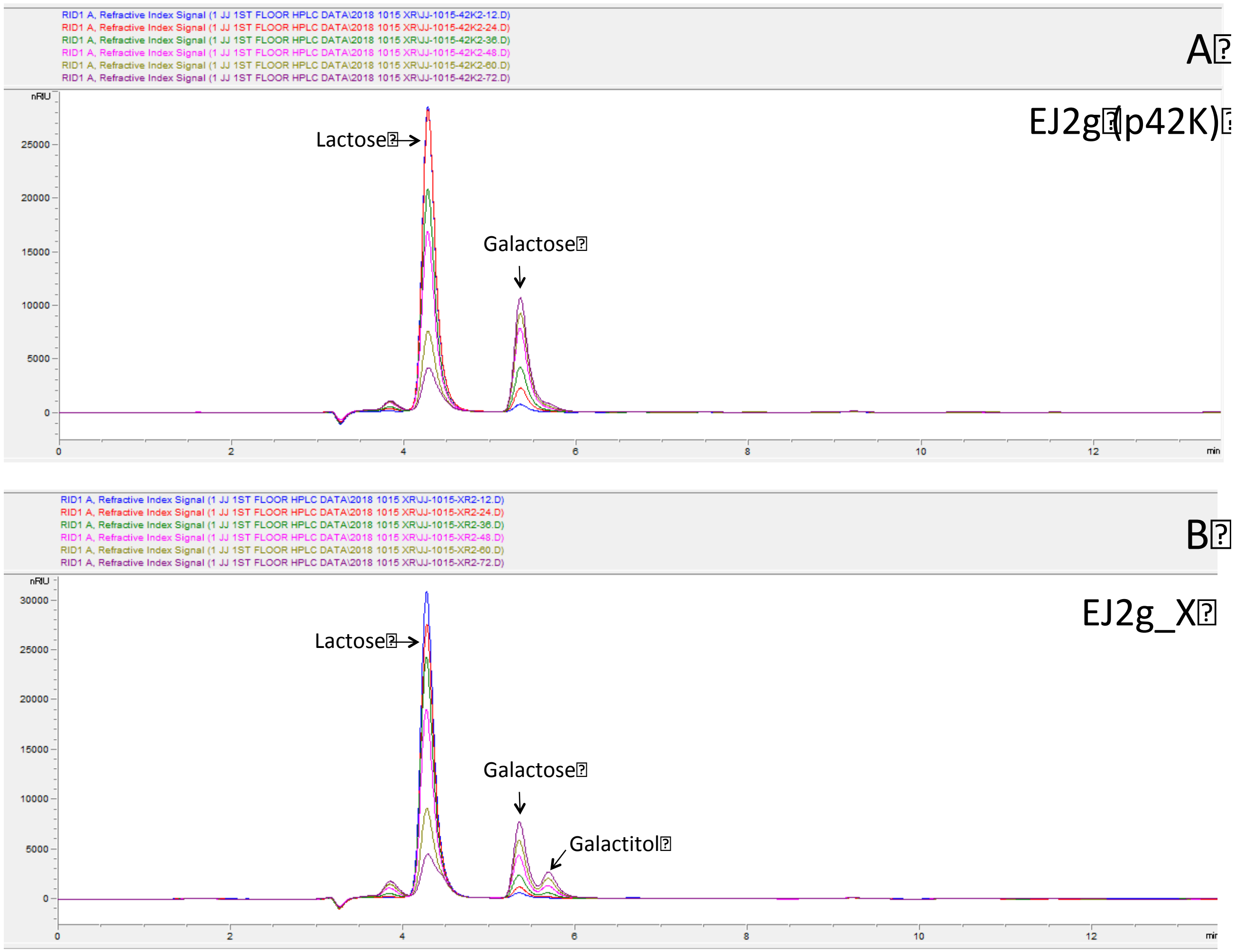

Supplementary Figure 2 HPLC chromatograms of the fermentation samples (A: EJ2g (p42K), B: EJ2g_pX) for Fig. 2. ROA-Organic Acid H+ column was used to separate lactose, galactose, and galactitol (one representative figure of triplicate samples during fermentation).

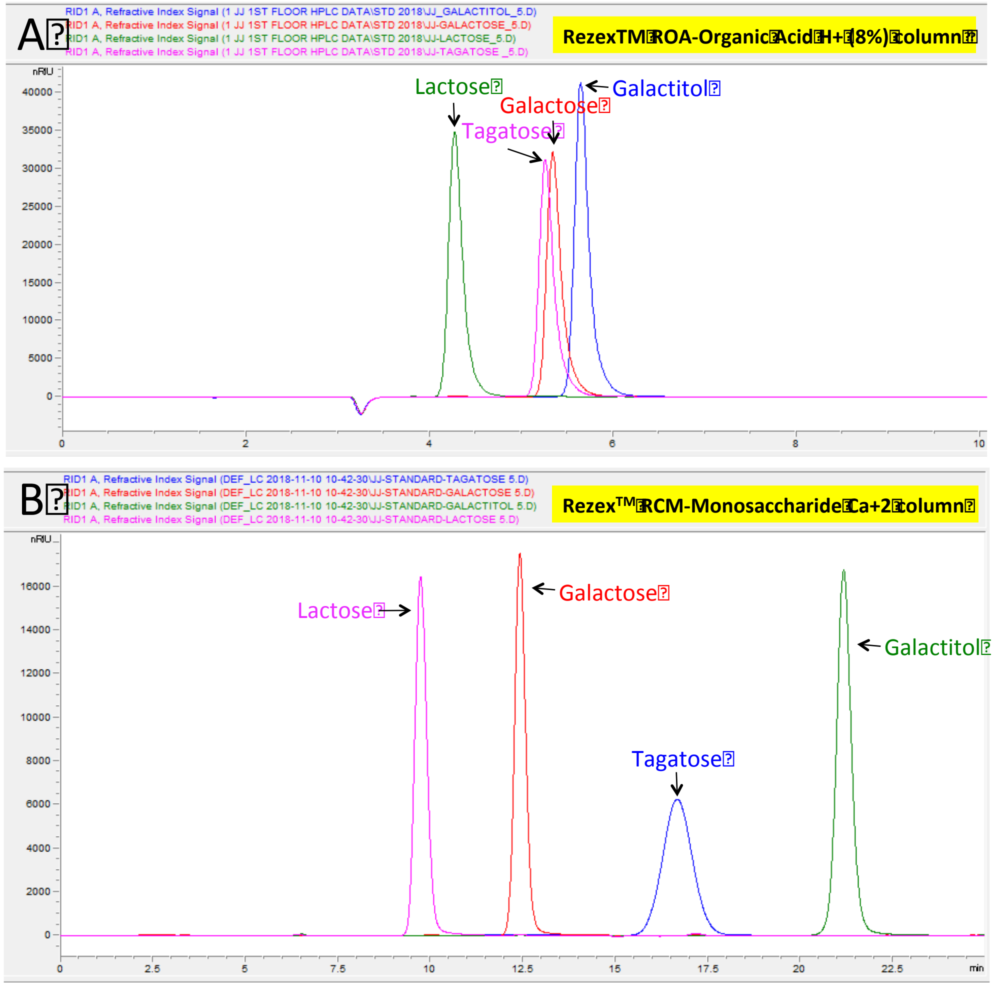

Supplementary Figure 3 The separation of galactose and tagatose by RCM-Monosaccharide Ca+2 column. While ROA-Organic Acid H+ column was not able to separate tagatose and glactose (A), RCM-Monosaccharide Ca+2 column separated tagatose and galactose well.

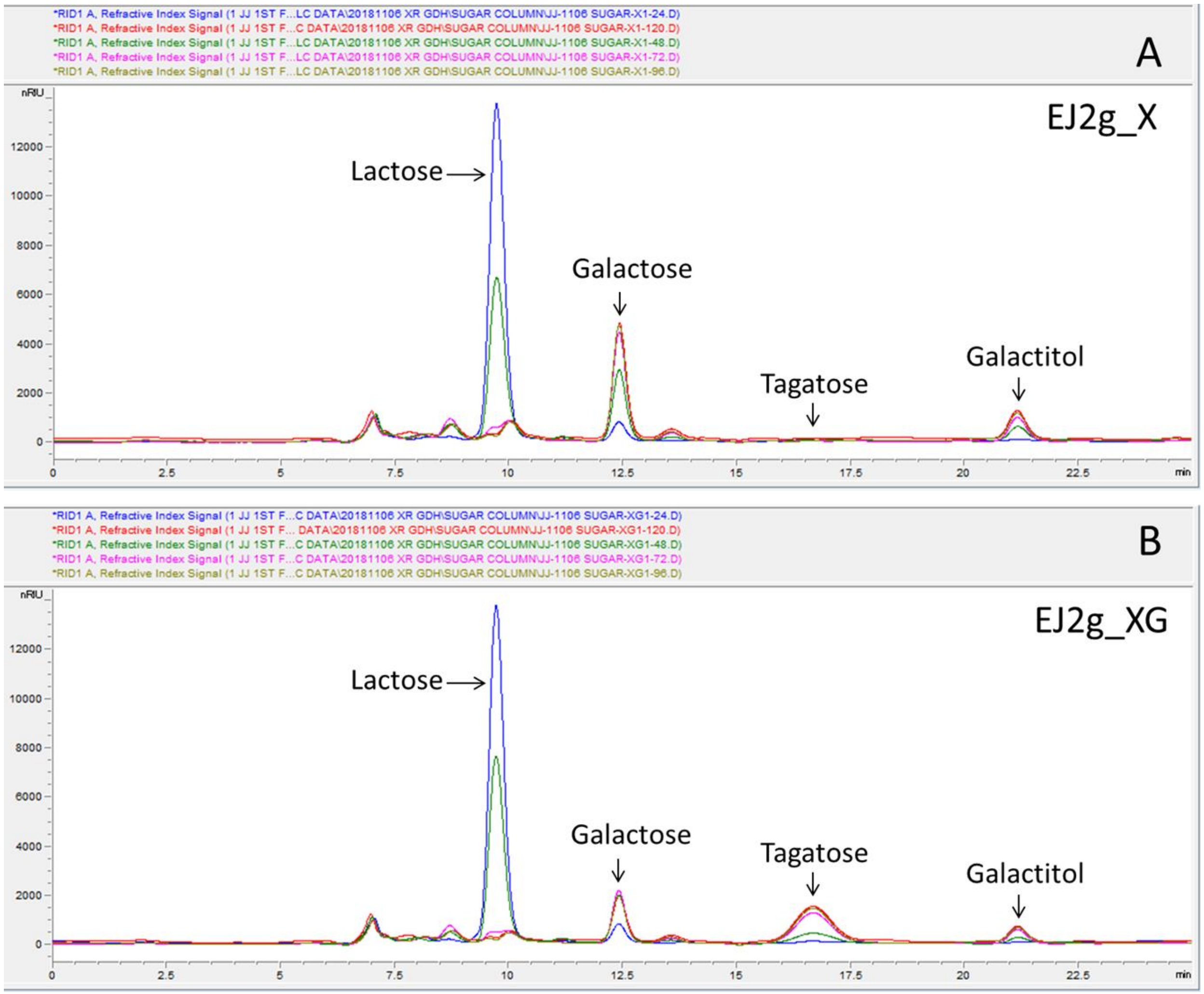

Supplmentary Figure 4 HPLC chromatograms of the fermentation samples (A: EJ2_pX, B: EJ2_pXpG) for Fig. 3 RCM-Monosaccharide Ca+2 column was used to separate lactose, galactose, tagatose, and galactitol.

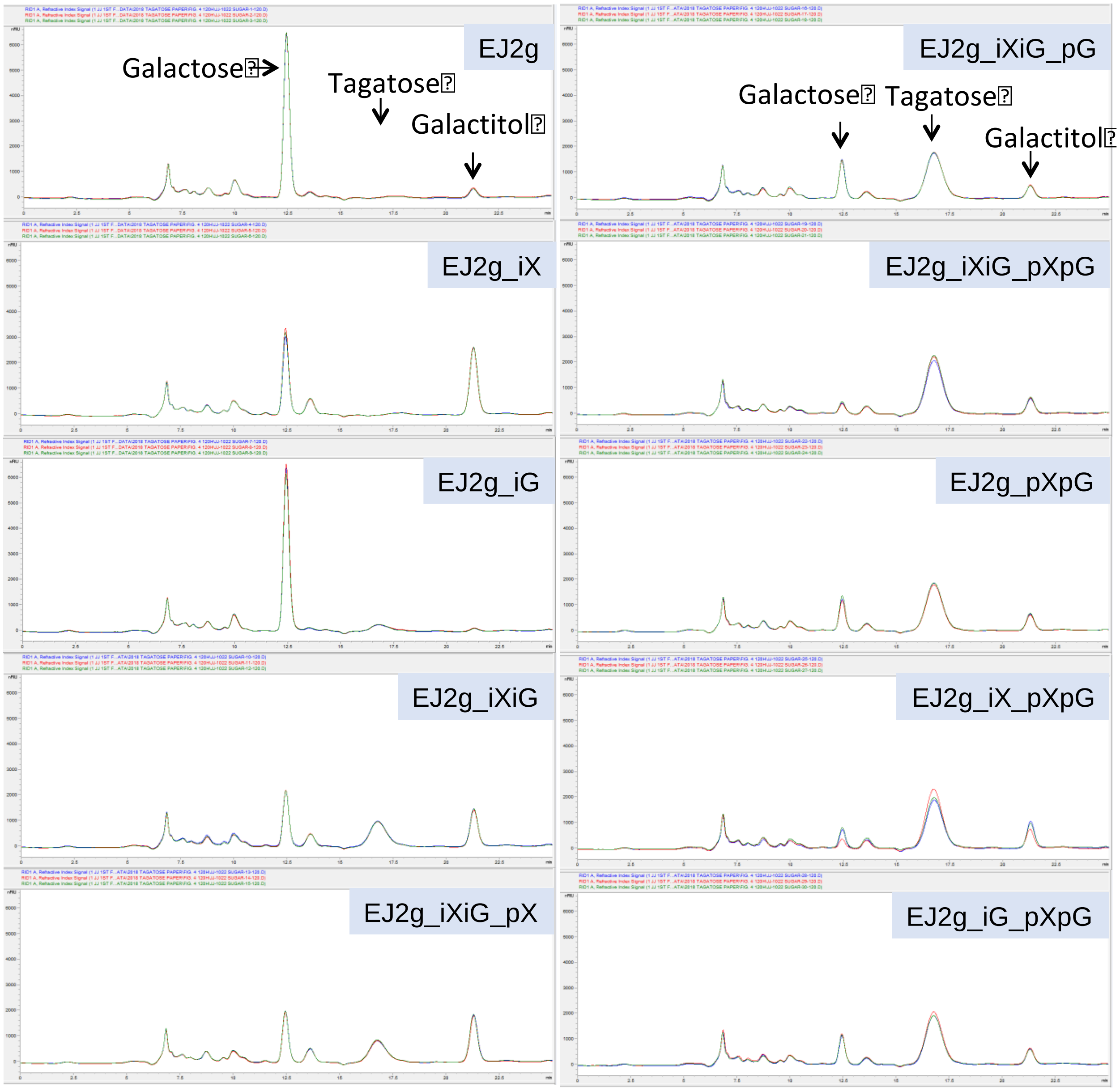

Supplementary Figure 5 HPLC chromatograms of the fermentation samples for Fig. 4 (triplicate samples were injected into RCM-Monosaccharide Ca+2 column was used to separate lactose, galactose, tagatose, and galactitol).

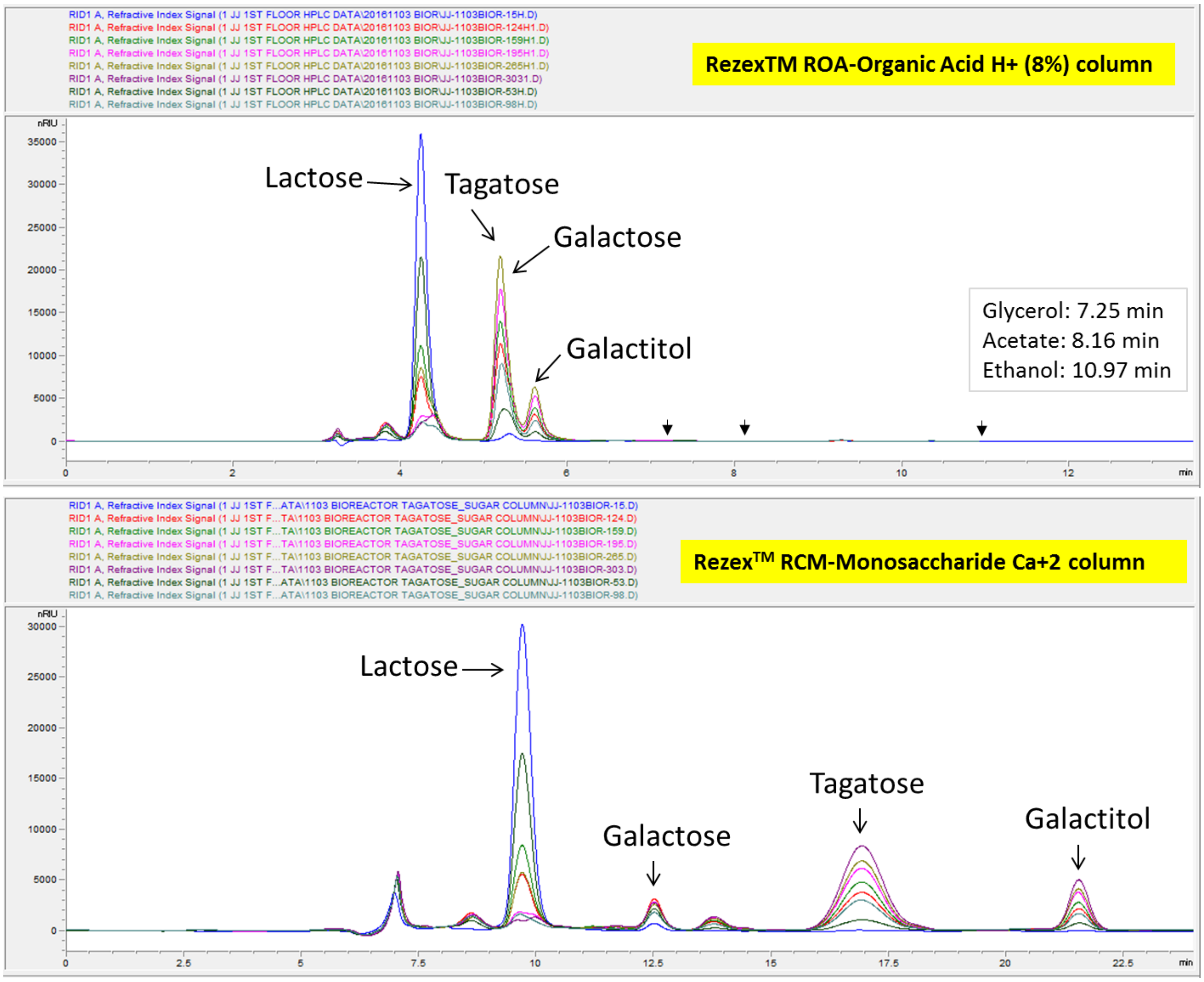

Supplementary Figure 6 HPLC chromatograms of the fermentation samples for Fig. 5 The samples of several time points of a bioreactor fermentation were injected into both ROA-Organic Acid H+ column and RCM-Monosaccharide Ca+2 column to separate lactose, tagatose, glactose, and galactitol as well as to detect glycerol, acetate, and ethanol. Neglibible amounts of glycerol, acetate, and ethanol were detected in the fermentation broth.

Supplementary References

1 Liu, J. J. *et al.* Metabolic Engineering of Probiotic Saccharomyces boulardii. *Appl Environ Microbiol* **82**, 2280-2287, doi:10.1128/AEM.00057-16 (2016).

2 Kwak, S. *et al.* Enhanced isoprenoid production from xylose by engineered *Saccharomyces cerevisiae*. *Biotechnol Bioeng* **114**, 2581-2591, doi:10.1002/bit.26369 (2017).

3 Taxis, C. & Knop, M. System of centromeric, episomal, and integrative vectors based on drug resistance markers for *Saccharomyces cerevisiae*. *Biotechniques* **40**, 73-78 (2006).

4 Jin, Y. S. & Jeffries, T. W. Changing flux of xylose metabolites by altering expression of xylose reductase and xylitol dehydrogenase in recombinant *Saccharomyces cerevisiae*. *Appl Biochem Biotechnol* **105 -108**, 277-286, doi:ABAB-106-1-3-277 [pii] (2003).

5 Mumberg, D., Muller, R. & Funk, M. Yeast vectors for the controlled expression of heterologous proteins in different genetic backgrounds. *Gene* **156**, 119-122, doi:0378111995000377 [pii] (1995).
