# Supplementary material for: Overcoming the thermodynamic equilibrium of an isomerization reaction through oxidoreductive reactions for biotransformation
Source: Nat Commun. 2019 Mar 22;10:1356. doi: 10.1038/s41467-019-09288-6 (PMC6430769; doi:10.1038/s41467-019-09288-6)
Supplement: Supplementary file 3 — Description of Additional Supplementary Files [file 41467_2019_9288_MOESM3_ESM.docx]

**Description of Additional Supplementary Files**

File Name: Supplementary Data 1

Description: Supplementary sequence information of the intergenic site CS6 for CRISPR-Cas9 based integration

File Name: Supplementary Data 2

Description: Supplementary sequence information of the intergenic site CS8 for CRISPR-Cas9 based integration
