# Supplementary material for: Overcoming the thermodynamic equilibrium of an isomerization reaction through oxidoreductive reactions for biotransformation
Source: Nat Commun. 2019 Mar 22;10:1356. doi: 10.1038/s41467-019-09288-6 (PMC6430769; doi:10.1038/s41467-019-09288-6)
Supplement: Supplementary file 2 — Peer Review File [file 41467_2019_9288_MOESM2_ESM.pdf]

Reviewers' comments:

Reviewer #1 (Remarks to the Author):

The manuscript by Liu et. al., entitled “Overcoming the thermodynamic equilibrium of an isomerization reaction through oxidoreductive reactions for biotransformation”, describes a novel method to overcome the equilibrium limitation in some enzymatic reactions, which uses a bypass metabolic pathway that harnesses redox reactions to favor the desired product. The authors demonstrate the potential of this approach in the production of tagatose, a desired sweetener, from galactose. Enabled by the ability of the cellobiose transporter (CDT-1) to transport lactose, and of β-glucosidase (GH1-1) to intracellularly hydrolyze lactose into glucose and galactose, the authors use a sugar reductase and dehydrogenase pair to convert galactose to tagatose. With this strategy, the authors achieve a tagatose:galactose ratio of 9:1, a remarkable improvement from the isomerase reaction (which achieves only a 3:7 ratio). This is an important study that will attract the interest of metabolic engineers, as well as researchers more broadly from biotechnology, enzyme catalysis, and biochemical engineering. Therefore, this reviewer supports its publication in Nature Communications.

Some minor revisions and clarifications are recommended:

1) What are the mechanisms of galactose, galactitol, and tagatose secretion? If the authors know or have some insight, it would benefit the manuscript to mention them and include them in their discussion.
2) It would be useful to spell out the loci and markers used to integrate genes in strains EJ2g_XG_X, _G, and _XG, so that the reader does not have to search previous publications to find out. This could be done in the Results, Methods, and/or Table III.
3) There is no mention of observed changes in the growth rates between the different strains. This is important because of the novelty of the carbon portioning strategy, as well as the potential effects of the accumulation of tagatose and its precursors, and ts of diverting redox cofactors towards tagatose formation of cell growth. The authors should show the growth rates og EJ2 and EJ2g, as well as derived strains EJ2g_XG_X, _G, and _XG. These strains have different rates of lactose consumption, and precursor accumulation so it seems likely they will also have differences in growth rates.
4) When the authors discuss the advantages of dividing the different monosaccharides derived from hydrolysis of a disaccharide into catabolic and anabolic pathways, they mention maltose and cellobiose as examples of potential substrates used in this approach (line 217). However, these disaccharides do not seem to be good expamples for carbon partinioning because they are both disaccharadies of glucose. Thus, their hydrolysis would produce two identical molecules of glucose, which would not be amenable to diverting into different anabolic/catabolic pathways.
5) A suggestion is to include in line 237: "...to increase yields, and further minimize the separation and purification cost."
6) Fig 4 is missing strains containing XR and GDH in plasmids plus either XR or GDH genomically integrated (EJ2g_XG_X, EJ2g_XG _G), even though these strains are mentioned in the paragraph that references this figure (lines 136-139. The data for these strains should be added to Fig 4.

These are all minor revisions. Once they are addressed, the study should be acceptable for publication in Nature Communications.

Reviewer #2 (Remarks to the Author):

The current manuscript elegantly describes how relatively simple strain engineering can be used to re-direct the fate of sugar substrates and turn them into commercially relevant products. It demonstrates how the weakness of end-reactions involving isomerases can in some cases be circumvented by a

two-step oxidoreductive pathway. These results and their conclusion will be interesting for both an academic and industrial reader

The manuscript is generally well written, and results are presented in an easy-to-follow and logical sequential way, although the discussion could ideally be shortened a bit to avoid repetition of points.

I note that the titres of tagatose in fig 6 keeps going up. I wonder how far that could go, and it will be interesting to see whether the presented strategy will be adopted by the sugar industry.

Comments to the main text:

L 104: Delete semi-colon

L 123: Maximum theoretical yield is mentioned here and several other times in the manuscript, but no calculation is presented (or reference). How was this calculated?

L 134: How many new strains were constructed, was it 4 or only 3 ?

L 134: I assume GDH instead of XDH?

L 134-42: The strain construction and the analysis of them does not correspond to what is shown in Figs 4 and 5. From the text you get the impression that integrations were done in EJ2g_XG, but I suspect it was actually done in EJ2g. This would explain the strains EJ2g X, EJ2g G, and EJ2g XG in Table III (which are not described elsewhere) and the number of strains analyzed in Figs 4 and 5.

A better explanation of construction, and of the set-up/choice of strains in Fig 4 and 5 is needed. In figs 4 and 5 it would be helpful to indicate, for each bar, the name of the strains analyzed. The names should be the same as used in the text. It would also help if the X on plasmid was named differently from the integrated X, e.g. by naming the latter one iX. The same for G and XG.

L 156: Remove semi-colon

L 181: Improve syntax

L 184: Check spelling of "co-factor" throughout manuscript and harmonize, see e.g. L 186

L 193-205: This section, in particular L 193-97, could be improved regarding language and clarity. As mentioned above, maybe the discussion as a whole could be tightened up.

L 247-49: The difference between YP + glucose and YPD is not clear

L 256: Donor instead of Donner in Gal1-Donnor… ? Same in Table I

L 272: Shaker amplitude? Not critical but information should be there

L 276: Rpm is relative depending on rotor (diameter, angle). What was the g-force?

L 281: Fig 6 A shows lactose levels oscillating between approx. 0-15 g/L. Please explain.

L 290: Ca+2 instead of Ca+2 ?

L 291: What is "EPure water" ?

Tables:

Table II: Is CS6 locus described anywhere? A reference perhaps?

Table III: See comment to L 134-42

Figures:

The legends are provided as two slightly different versions (at least in the PDF I am reviewing). Which one is going to be used?

Fig 1,2, and 3: Suggest to harmonize legends with respect to the order in which you refer to figures, and the use of parenthesis. The varies from figure to figure

Fig 4 and 5: It is not easy to decipher which strains are referred to, and it might be better to use the same naming as used in the main text. Maybe you could indicate integration by a lower case "i" for example: EJ2g_XG_iX and EJ2g_XG_iXiG (same in Table III). See also comment to L134-42. In order to include strain names it may be an option to present results as horizontal bars? Or use numbers/letters to name bars, and use legend to explain which strains they are.

Fig 6: In the legend, assume XR_XR should be XG_XG (or XG_iXiG, as suggested by this reviewer :-)

Reviewer #3 (Remarks to the Author):

This manuscript was described on the production of tagatose from lactose with a ratio exceeding the thermodynamic limit of an isomerization of tagatose to galactose using an engineered yeast. This manuscript shows some novelty on the production of tagatose from lactose with a high ratio of tagatose to galactose and is of interest to readers in the field. However, the manuscript cannot be accepted because of many critical problems.

General comments

1. Authors claimed that the ratio of tagatose to galactose produced by engineered yeast expressing xylose reductase (XR) and galactitol 2-dehydrogenase (GDH) in the fermentation broth was 9:1. The reaction products by L-arabinose isomerase are galactose and tagatose, whereas the fermentation broth contained not only galactose and tagatose but also lactose, ethanol, galactitol, and other unidentified metabolites. Moreover, GDH converted galactitol into tagatose with a conversion ratio of 72% (Reference #16) and Enterobacter agglomerans strain 221e converted galactitol into tagatose with a conversion ratio of 86% by the one-step reaction (Reference: Muniruzzanman, S. et al. J. Ferment. Bioeng. 78, 145-148 (1994).). Engineered yeast in this study converted galactose into tagatose via galactitol by the two-step reaction with higher ratio (a conversion ratio of 90%) than the one-step reaction. This result is not reasonable. Thus, the ratio of 9:1 is not exact. For the determination of the exact ratio, whole cell reaction using the harvested cells of engineered yeast should be performed with galactose, not lactose, to produce tagatose.

2. This manuscript seemed to exhibit novelty on the production of tagatose from galactose by engineered yeast with XR and GDH and on carbon partition strategy, glucose consumption and tagatose production by engineered yeast. As described above references, GDHs have been applied to tagatose production. Carbon partition strategy was already used for tagatose production (Wanarska, M. & Kur, J. A method for the production of D-tagatose using a recombinant Pichia pastoris strain secreting β-D-galactosidase from Arthrobacter chlorophenolicus and a recombinant L-arabinose isomerase from Arthrobacter sp. 22c. Microb. Cell Fact. 11,113 (2012).) Thus, the novelty of the

manuscript was not critically improved, compared with those of the previous reports.

3. Engineered yeast produced 37.7 g/L tagatose from lactose for 300 h with a productivity of 0.126 g/L/h. L-Arabinose isomerase converted 500 g/L galactose into 370 g/L tagatose for 24 h in the presence of boric acid with a productivity of 15.4 g/L/h, and converted 300 g/L galactose into 158 g/L tagatose for 20 h in the absence of boric acid (Reference, Lim, B. C. et al. Biotechnol. Prog. 23, 824-828 (2007).). The concentration and productivity of tagatose in this study were 10- and 120-fold lower than the highest reported values. Thus, the results in this study are too low concentration and productivity. The improvement of the concentration and productivity tagatose is required.

4. The manuscript does not provide strong evidence for its conclusions, the data are not technically sound, and more exact and fair comparison with other reports is needed.

5. The disadvantages of the fermentation method used in this study for purification were not described. The galactose and tagatose of the enzyme reaction solution were easily separated using the moving-bed chromatography system because it contained little other products. The fermentation both contained medium, byproducts (lactose, ethanol, and galactitol), color, and other unidentified metabolites. Especially, the purification of tagatose is difficult when ethanol is present. Therefore, the purification of tagatose from the fermentation both is difficult and it requires high cost.

6. The explanation for the comparison of plasmids (the section of 'Intracellular conversion of galactose into tagatose by oxidoreductases') was not clear and was not understood. Moreover, the used types of plasmids were too few.

7. The concepts between 'equilibrium ratio' and 'ratio' were not clear. Authors claimed that the ratio of galactose and tagatose is around 7:3. The ratio of galactose and tagatose is 7:3 at 30 °C and around 5:5 at 60 °C. However, the production of tagatose by L-arabinose isomerase has been almost performed at 60-65 °C.

8. The many content of 'Introduction' was the same as that of 'Discussion'. Please remove the repeated contents.

9. Please, check 'Guide to authors'. For example, an abstract contains approximately 150 words according to the guide. However, this manuscript contained approximately 250 words. Reduce the words in the abstract. Moreover, the abstract should be intensively revised because of inaccurate expression. The primer design in Table I is not important. Transfer it to Supplementary data.

9. The citation is limited. I suggest the citation of more diverse and exact References. Moreover, in Reference section, there were too many spelling errors and format errors. References should be revised point by point.
1) All journal names of references should be revised. For examples, the journal name of 'J Agric Food Chem' should be revised to 'J. Agric. Food Chem.'. The journal name of 'Nutrition and Food Sciences Research' should be revised to 'Nutr. Food Sci. Res.'.
2) Reference numbers 3-6, 11, 14-17, 19, 21-26, and 29: The names of microorganisms should be italicized.
3) Reference numbers 3-6, 10-13, 15, 16, 21-23, 25, and 27: The words of 'L-', 'D-',' 'l-', and 'd-' should be revised to the small capital 'L-', and 'D-'.
4) Reference numbers 2, 17, and 30: Title should be small letters except for the first word.
5) Reference 1: What is the meaning for 'US$ 183.50 (hardback)'.

Specific comments

1. Abstract: Please, include the lactose-related contents because the key word is ‘lactose’

2. Abstract, L28, L41: The ratio of ‘3:7’ is not exact value.

3. Abstract, L31: Authors described that the oxidoreductases require cofactors. Thus, provide the solution for the requirement of cofactors.

4. Abstract, L38-39: How do you perform that “the expression levels of XR and GDH were adjusted to optimize tagatose production”

5. Abstract, L40: Please, insert the term of ‘in the reaction broth’ after ‘with a ratio of 9:1’. Engineered yeast produced 37.7 g/L of tagatose from not galactose but lactose. The ratio of ‘9:1’ is not exact value because the reaction broth contained lactose, ethanol, and galactitol. The ratio should be tagatose per galactose, lactose, ethanol, and galactitol.

6. Abstract, L46: Remove the word of ‘L-arabinose isomerase’ and insert the word of ‘galactose’.

7. Introduction, L76-78: The explanation of the sentence is not correct because tagatose production was performed by not enzyme but immobilized cells.

8. Introduction, L84: The ratio of ‘7:3’ is not exact value. I suggest ‘5:5’.

9. Introduction, L86: The word of ‘S. cerevisiae’ should be revised to ‘Saccharomyces cerevisiae’.

10. Results, L104: The HPLC results of Fig. 1 should be provided in Supplementary data.

11. Results, L116-119: NADPH-linked XR was coupled with NAD+-linked GDH but not NADP+-linked GDH. Explain the regeneration of cofactors.

12. Results, L118-119: The HPLC results of Fig. 2 should be provided in Supplementary data.

13. Results, L123: The HPLC results of Fig. 3 should be provided in Supplementary data.

14. Results, L136-137: Please, explain more detail for EJ2g_XG_X, EJ2g_XG_G, and EJ2g_XG_XG.

15. Results, L145: The difference between the yield of tagatose from lactose by EJ2g_XG_XG and the theoretical maximum yield should be described.

16. Results, L150-152: Please, described the total amount of lactose (g/L) at the fed-batch fermentation.

17. Results, L157: The HPLC results of Fig. 6 at several fermentation times should be provided in Supplementary data.

18. Results, L157: The ratio of ‘3:7’ is not exact value. I suggest ‘5:5’.

19. Results, L142: Please, explain the content of Fig. 5.

20. Results, 147-162: The experiments for tagatose production with increasing the total amount of lactose at the fed-batch fermentation should be performed.

21. Results, 156: The ratio of ‘9:1’ is not exact value because the reaction broth contained lactose, ethanol, and galactitol. The ratio should be tagatose per galactose, lactose, ethanol, and galactitol.

22. Discussion, 170-174: I suggest ‘L-arabinose isomerase’ instead of xylose isomerase.

23. Discussion, 178-182, 227-228: The explanation of the sentence is not correct because tagatose production was performed by not enzyme but immobilized cells. The separation process using enzyme reaction is simpler than that using fermentation.

24. Materials and Methods, 244: Describe yeast strain.

25. Materials and Methods, 246: The word of ‘Ampicillin’ should be revised to ‘ampicillin’.

26. Materials and Methods, 244: Cell mass should determined using a calibration curve derived from the correlation between the dry cell weight and the OD600 of yeast fermentation broth.

27. Tables: Please, transfer Table I and Table II to Supplementary data.

28. Figures:
1) In the all figures, fermentation conditions and method should be included.
2) Combine Fig. 1 and Fig. 2.
3) Please, transfer Fig. 5 to Supplementary data because the explanation in the text is enough.
4) The term of ‘~30%’ in Fig. 5 and its legend should be revised to ‘~50%’.
5) The references in Fig. 5 should be changed to those showing ‘~50%’.
6) The OD600 in Fig. 6B should be revised to dry cell weight (g/L).

Reviewer #1 (Remarks to the Author):

The manuscript by Liu et. al., entitled "Overcoming the thermodynamic equilibrium of an isomerization reaction through oxidoreductive reactions for biotransformation", describes a novel method to overcome the equilibrium limitation in some enzymatic reactions, which uses a bypass metabolic pathway that harnesses redox reactions to favor the desired product. The authors demonstrate the potential of this approach in the production of tagatose, a desired sweetener, from galactose. Enabled by the ability of the cellobiose transporter (CDT-1) to transport lactose, and of β-glucosidase (GH1-1) to intracellularly hydrolyze lactose into glucose and galactose, the authors use a sugar reductase and dehydrogenase pair to convert galactose to tagatose. With this strategy, the authors achieve a tagatose:galactose ratio of 9:1, a remarkable improvement from the isomerase reaction (which achieves only a 3:7 ratio). This is an important study that will attract the interest of metabolic engineers, as well as researchers more broadly from biotechnology, enzyme catalysis, and biochemical engineering. Therefore, this reviewer supports its publication in Nature Communications.

Some minor revisions and clarifications are recommended:

1) What are the mechanisms of galactose, galactitol, and tagatose secretion? If the authors know or have some insight, it would benefit the manuscript to mention them and include them in their discussion.
Our response: Thank you for providing the constructive comment. The mechanisms of galactose, galactitol, and tagatose secretion are not well known. However, we speculate that secretion of the sugar and sugar alcohol might be facilitated by sugar transporters or glycerol transporter in yeast. Specifically, Gal2 (galactose permease) might be responsible for the secretion and re-assimilation of galactose, and Fps1 (aquaglyceroporin), which have been reported to transport xylitol (Wei et al., 2013), might be involved in the secretion of galactitol. As Gal2 might be involved in both secretion and re-assimilation of galactose, up-regulation of *GAL2* might increase the secretion and re-assimilation of galactose, and deletion might reduce the secretion and re-assimilation of galactose. As such, the effects of *GAL2* perturbation will be mixed or compromised. For Fps1, since galactitol is structurally similar to xylitol, the deletion of *FPS1* in a tagatose producing strain might lead to the reduced production of galactitol. As the reviewer suggested, we added the following paragraph into Discussion (L192-206).

(L192-206) "While direct and efficient conversion of lactose into tagatose by the oxidoreductase pathway has been demonstrated in this study, the secretion of galactose and galactitol during the conversion needs to be addressed in order to reach the theoretical maximum yield. We speculate that the secretion of galactose and galactitol might be facilitated by endogenous hexose and sugar alcohol transporter in yeast. Specifically, Gal2 (galactose permease) might be responsible for the secretion and re-assimilation of galactose, and Fps1 (aquaglyceroporin), which have been reported to transport xylitol [32], might be involved in the secretion of galactitol. From the structural similarity between galactitol and xylitol, the deletion of *FPS1* in a tagatose producing strain might reduce production of galactitol. As Gal2 might be involved in both secretion and re-assimulation of galactose, up-regulation of *GAL2* might increase the secretion and re-assimilation of galactose, and deletion might reduce the secretion and re-assimilation of

galactose. As such, the effects of *GAL2* perturbation would likely be mixed or compromised. Genetic perturbations of endogenous sugar and sugar alcohol transporters including *FPS1* and *GAL2* can be conducted in the future to further improve the conversion yield of tagatose from lactose."

2) It would be useful to spell out the loci and markers used to integrate genes in strains EJ2g_XG_X, _G, and _XG, so that the reader does not have to search previous publications to find out. This could be done in the Results, Methods, and/or Table III.

Our response: Thanks for the suggestion. We changed the notation of the strains systematically and used the new names consistently in the Results, Method, and Tables. The changes are summarized in the following table. X and G denote XR and GDH and supercripts i and p denote integrated and plasmid copies, respectively.

Strains	Previous name	New name
1	EJ2g X	EJ2g_iX
2	EJ2g G	EJ2g_iG
3	EJ2g XG	EJ2g_iXiG
4	EJ2g_XG_X	EJ2g_iXiG_pX
5	EJ2g_XG_G	EJ2g_iXiG_pG
6	EJ2g_XG_XG	EJ2g_iXiG_pXpG

We also added detailed information about the gene integration loci of CS6 and CS8 in Methods as suggested (L272-288).

L272-288, "The CS6 and CS8 sites are selected intergenic regions for integration of expression cassettes via Cas9-based genome editing. The CS6 site is located between YGR190C and tW(CCA)G2 in Chromosome VII, and the CS8 is located between YPR014C and YPR015C in Chromosome XVI. Targeting guide RNA sequences for CS6 and CS8 are listed in Table S2. Detailed sequence information of the CS6 and CS8 sites are provided in Supplementary Information. Based on the targeting sequences, guide RNA expressing plasmids (p42K-CS8 and p42K-CS6) were constructed by reverse PCR of a pRS42K plasmid containing guide RNA sequence using primer pairs gCS6-U/gCS6-D and gCS8-U/gCS8-D, respectively (Table S1) [39]. For genomic integration of *XYL1* into the EJ2g strain, the plasmid p42K-XR were amplified using a primer pair of CS6-IU and CS6-ID as donor DNAs for CRISPR-Cas9 based integration. The transformants with CS6-XR integration were confirmed by PCR using primers CS6-CKU and CS6-CKD, and was designated as the EJ2g_iX strain (Table 1). Similarly, CS8-GDH was integrated into the EJ2g_iX strain with a donor DNA amplified from plasmid p42H-GDH using primers CS8-IU and CS8-ID. The transformants were confirmed by PCR using primers CS8-CKU and CS8-CKD and the engineered strain with CS6-XR and CS8-GDH integration was named as the EJ2g_iXiG strain (Table 1)."

3) There is no mention of observed changes in the growth rates between the different strains. This is important because of the novelty of the carbon portioning strategy, as well as the potential effects of the accumulation of tagatose and its precursors, and ts of diverting redox cofactors towards tagatose formation of cell growth. The authors should show the growth rates

og EJ2 and EJ2g, as well as derived strains EJ2g_XG_X, _G, and _XG. These strains have different rates of lactose consumption, and precursor accumulation so it seems likely they will also have differences in growth rates.
Our response: We did not measure growth curves previously because we were mainly focusing on the sugar conversion. However, we agree that the reviewer raised a good point. In the revised manuscript, we repeated the fermentation experiments using the strains and added the growth curves into Fig. 1, Fig. 2, and Fig. 3.

4) When the authors discuss the advantages of dividing the different monosaccharides derived from hydrolysis of a disaccharide into catabolic and anabolic pathways, they mention maltose and cellobiose as examples of potential substrates used in this approach (line 217). However, these disaccharides do not seem to be good expamples for carbon partinioning because they are both disaccharadies of glucose. Thus, their hydrolysis would produce two identical molecules of glucose, which would not be amenable to diverting into different anabolic/catabolic pathways.
Our response: We agree that maltose and cellobiose cannot be used for carbon partitioning strategy as they consist of glucose only. We deleted maltose and cellobiose in our revised manuscript.

5) A suggestion is to include in line 237: "...to increase yields, and further minimize the separation and purification cost."
Our response: Thank you. We changed the sentence based on the suggestion (Line 244).

6) Fig 4 is missing strains containing XR and GDH in plasmids plus either XR or GDH genomically integrated (EJ2g_XG_X, EJ2g_XG _G), even though these strains are mentioned in the paragraph that references this figure (lines 136-139. The data for these strains should be added to Fig 4.
Our response: We added the fermentation profiles of the missing strains (EJ2g_XG_X; new name EJ2g_iXiG_pX and EJ2g_XG_G; new name EJ2g_iXiG_pG) as suggested into Fig. 4.

These are all minor revisions. Once they are addressed, the study should be acceptable for publication in Nature Communications.
Our response: We sincerely appreciate the reviewer for his/her constructive comments.

Reviewer #2 (Remarks to the Author):

The current manuscript elegantly describes how relatively simple strain engineering can be used to re-direct the fate of sugar substrates and turn them into commercially relevant products. It demonstrates how the weakness of end-reactions involving isomerases can in some cases be circumvented by a two-step oxidoreductive pathway. These results and their conclusion will be interesting for both an academic and industrial reader

The manuscript is generally well written, and results are presented in an easy-to-follow and logical sequential way, although the discussion could ideally be shortened a bit to avoid repetition of points.

I note that the titres of tagatose in fig 6 keeps going up. I wonder how far that could go, and it will be interesting to see whether the presented strategy will be adopted by the sugar industry.
Our response: We really appreciate the reviewer for the positive and encouraging comments. We agree with the reviewer that scale up tagatose production for the real industrial fermentation is interesting. We are open to collaborate with an industrial partner to commercialize our fermentation process.

Comments to the main text:

L 104: Delete semi-colon
Our response: We deleted semi-colon as suggested.

L 123: Maximum theoretical yield is mentioned here and several other times in the manuscript, but no calculation is presented (or reference). How was this calculated?
Our response: We added the calculation in Materials and Methods (L319-322).

L319-322, "The maxium theoretical tagatose yield (0.526 g tagatose/g lactose) by engineered yeast was calculated based on the molecular weights of tagatose (180.16 g/mol) and lactose (342.3 g/mol) with an assumption that glucose is not coverted into tagatose."

L 134: How many new strains were constructed, was it 4 or only 3 ?
Our response: Sorry for the confusion. We constructed 3 more strains. We made changes accordingly in our revised manuscript.

L 134: I assume GDH instead of XDH?
Our response: Yes, you are correct. We changed "XDH" into "GDH" in our revised manuscript.

L 134-42: The strain construction and the analysis of them does not correspond to what is shown in Figs 4 and 5. From the text you get the impression that integrations were done in EJ2g_XG, but I suspect it was actually done in EJ2g. This would explain the strains EJ2g X, EJ2g G, and EJ2g XG in Table III (which are not described elsewhere) and the number of strains analyzed in Figs 4 and 5.

A better explanation of construction, and of the set-up/choice of strains in Fig 4 and 5 is needed. In figs 4 and 5 it would be helpful to indicate, for each bar, the name of the strains analyzed. The names should be the same as used in the text. It would also help if the X on plasmid was named differently from the integrated X, e.g. by naming the latter one iX. The same for G and XG.
Our response: Thanks for the suggestion. We renamed our strains as suggested. The changes are summarized in the following table. X and G denote XR and GDH and prefix i and p denote integrated and plasmid copies, respectively.

Strains	Previous name	New name
1	EJ2g X	EJ2g_iX
2	EJ2g G	EJ2g_iG

3	EJ2g XG	EJ2g_iXiG
4	EJ2g_XG_X	EJ2g_iXiG_pX
5	EJ2g_XG_G	EJ2g_iXiG_pG
6	EJ2g_XG_XG	EJ2g_iXiG_pXpG

L 156: Remove semi-colon
Our response: We deleted the semi-colon as suggested.

L 181: Improve syntax
Our response: We have rearranged the text in our revised manuscript.

L 184: Check spelling of "co-factor" throughout manuscript and harmonize, see e.g. L 186
Our response: We changed "co-factor" into "cofactor" in our revised manuscript.

L 193-205: This section, in particular L 193-97, could be improved regarding language and clarity. As mentioned above, maybe the discussion as a whole could be tightened up.
Our response: The discussion section has been tightened up and revised by native speakers.

L 247-49: The difference between YP + glucose and YPD is not clear
Our response: Sorry for the confusion. They are the same. We used YPD only.

L 256: Donor instead of Donner in Gal1-Donnor… ? Same in Table I
Our response: Yes, the reviewer is correct. We corrected it.

L 272: Shaker amplitude? Not critical but information should be there
Our response: We added detailed info: New Brunswick™ Innova® 2300 shaker with 250 rpm and orbit of 2.5 cm.

L 276: Rpm is relative depending on rotor (diameter, angle). What was the g-force?
Our response: The g-force is 15294 g.

L 281: Fig 6 A shows lactose levels oscillating between approx. 0-15 g/L. Please explain.
Our response: We found that high concentration of lactose is toxic to the cell and they will produce acetate acid which will inhibit tagatose production. Thus, we maintained lactose levels below 20 g/L through pulse feeding in order to avoid acetate acid production.

L 290: Ca+2 instead of Ca+2 ?

Our response: Ca+2 is a part of RezexTM RCM-Monosaccharide Ca+2 (8%) column name.

L 291: What is "EPure water" ?
Our response: We changed "EPure water" into "E-pure™ water (Barnstead E-pure™ Water Purification Systems, Thermo Scientific)" to clarify. This is purified water from Barnstead E-pure™ Water Purification Systems (https://us.vwr.com/store/product/4952829/barnstead-e-pure-water-purification-systems-thermo-scientific).

Tables:

Table II: Is CS6 locus described anywhere? A reference perhaps?
Our response: We added the description (L272-288) of the CS6 locus in the Material and Method and Supplementary Information. CS6 locus was already reported and we have added the reference (Kwak, Kim et al. 2017) in our revised manuscript.

Table III: See comment to L 134-42
Our response: We have renamed the strains systematically.

Figures:

The legends are provided as two slightly different versions (at least in the PDF I am reviewing). Which one is going to be used?
Our response: We made the legends consistent in our revised manuscript.

Fig 1,2, and 3: Suggest to harmonize legends with respect to the order in which you refer to figures, and the use of parenthesis. The varies from figure to figure
Our response: We made the legends consistent in our revised manuscript.

Fig 4 and 5: It is not easy to decipher which strains are referred to, and it might be better to use the same naming as used in the main text. Maybe you could indicate integration by a lower case "i" for example: EJ2g_XG_iX and EJ2g_XG_iXiG (same in Table III). See also comment to L134-42. In order to include strain names it may be an option to present results as horizontal bars? Or use numbers/letters to name bars, and use legend to explain which strains they are.
Our response: Thanks for the suggestion. We renamed the strains as suggested and made a horizontal bar chart (Fig. 4) with new strains names as suggested. It is now easier to read.

Fig 6: In the legend, assume XR_XR should be XG_XG (or XG_iXiG, as suggested by this reviewer :-)
Our response: Thank you. We corrected the name of the strain as suggested.

Reviewer #3 (Remarks to the Author)

This manuscript was described on the production of tagatose from lactose with a ratio exceeding the thermodynamic limit of an isomerization of tagatose to galactose using an engineered yeast. This manuscript shows some novelty on the production of tagatose from lactose with a high ratio

of tagatose to galactose and is of interest to readers in the field. However, the manuscript cannot be accepted because of many critical problems.

General comments

1. Authors claimed that the ratio of tagatose to galactose produced by engineered yeast expressing xylose reductase (XR) and galactitol 2-dehydrogenase (GDH) in the fermentation broth was 9:1. The reaction products by L-arabinose isomerase are galactose and tagatose, whereas the fermentation broth contained not only galactose and tagatose but also lactose, ethanol, galactitol, and other unidentified metabolites. Moreover, GDH converted galactitol into tagatose with a conversion ratio of 72% (Reference #16) and Enterobacter agglomerans strain 221e converted galactitol into tagatose with a conversion ratio of 86% by the one-step reaction (Reference: Muniruzzanman, S. et al. J. Ferment. Bioeng. 78, 145-148 (1994).). Engineered yeast in this study converted galactose into tagatose via galactitol by the two-step reaction with higher ratio (a conversion ratio of 90%) than the one-step reaction. This result is not reasonable. Thus, the ratio of 9:1 is not exact. For the determination of the exact ratio, whole cell reaction using the harvested cells of engineered yeast should be performed with galactose, not lactose, to produce tagatose.
Our response: The ratio of galactose and tagatose at the end of fermentation was indeed 1:9 in the final sample from the bioreactor (we included the HPLC chromatograms of the samples in the Supplementary Information Fig. S6). The reactions were driven by favorable intracellular concentrations of NADPH and NAD^+ so that we could achieve high conversion yield through yeast fermentation. Similar conversion of xylose into xylulose in engineered yeast allows near 100% conversion when coupled with a subsequent xylulokinase reaction (Kim, Ha et al. 2012, Kim, Park et al. 2013, Kim, Skerker et al. 2013, Oh, Ha et al. 2013). We think that use of lactose is important because the consumption of glucose under aerobic conditions produces surplus NADPH and NAD^+ to drive the redox reactions.

2. This manuscript seemed to exhibit novelty on the production of tagatose from galactose by engineered yeast with XR and GDH and on carbon partition strategy, glucose consumption and tagatose production by engineered yeast. As described above references, GDHs have been applied to tagatose production. Carbon partition strategy was already used for tagatose production (Wanarska, M. & Kur, J. A method for the production of D-tagatose using a recombinant Pichia pastoris strain secreting β-D-galactosidase from Arthrobacter chlorophenolicus and a recombinant L-arabinose isomerase from Arthrobacter sp. 22c. Microb. Cell Fact. 11,113 (2012).) Thus, the novelty of the manuscript was not critically improved, compared with those of the previous reports.
Our response: We believe (and Reviewers 1 and 2 agreed) that this manuscript is novel in the fact that the beta-glucosidase used is an intracellular enzyme, bypassing glucose repression problem, and allowing glucose to be utilized as tagatose is being produced. This is different from Wanarska et al., which used secreted beta-glucosidase. Wanarksa et al. also did not directly address the unfavorable reaction equilibria as they employed an isomerase enzyme.

3. Engineered yeast produced 37.7 g/L tagatose from lactose for 300 h with a productivity of 0.126 g/L/h. L-Arabinose isomerase converted 500 g/L galactose into 370 g/L tagatose for 24 h

in the presence of boric acid with a productivity of 15.4 g/L/h, and converted 300 g/L galactose into 158 g/L tagatose for 20 h in the absence of boric acid (Reference, Lim, B. C. et al. Biotechnol. Prog. 23, 824-828 (2007).). The concentration and productivity of tagatose in this study were 10- and 120-fold lower than the highest reported values. Thus, the results in this study are too low concentration and productivity. The improvement of the concentration and productivity tagatose is required.

Our response: The referred work used 10-fold high starting concentration of galactose in comparison to our lactose starting material, hence the natural difference in tagatose productivity in g/L/h unit. When comparing our tagatose yield 62.73% to their yield 74%, it is quite comparable. Additionally, our strategy which allows utilization of lactose is much more economically favorable as lactose is commercially available and priced at $910/ton (https://www.globaldairytrade.info/en/product-results/lactose/), while galactose needs to be derived from lactose, a process that incurs additional costs. Lastly, boric acid (not allowable in food ingredients) is not easy to separate from the mixture.

4. The manuscript does not provide strong evidence for its conclusions, the data are not technically sound, and more exact and fair comparison with other reports is needed.

Our response: We presented a new concept of producing a sugar isomer (tagatose) through carbon partition and oxidoreductive enzymes instead of conventional isomerase reactions which often suffer from unfavorable reaction equilibria. Direct and fair comparison of our results with other results based on enzymatic reactions might not be possible as substrate and reaction conditions are quite different.

5. The disadvantages of the fermentation method used in this study for purification were not described. The galactose and tagatose of the enzyme reaction solution were easily separated using the moving-bed chromatography system because it contained little other products. The fermentation both contained medium, byproducts (lactose, ethanol, and galactitol), color, and other unidentified metabolites. Especially, the purification of tagatose is difficult when ethanol is present. Therefore, the purification of tagatose from the fermentation both is difficult and it requires high cost.

Our response: Only negligible amounts of ethanol, acetate, and glycerol production were detected during the fermentations by our engineered strains. Please look into chromatograms of both sugar and organic acid analysis columns (Figs S2 and S6). We expect that galactose and galactitol secretion can be minimized by further strain improvements. We are not experts of downstream purification process after fermentation but we do not expect outstanding difficulties as sugar purification and refining are near matured technologies. Solubility of galactitol is low and we can separate it easily from the fermentation broth. Galactose could be consumed by wild type yeast.

6. The explanation for the comparison of plasmids (the section of 'Intracellular conversion of galactose into tagatose by oxidoreductases') was not clear and was not understood. Moreover, the used types of plasmids were too few.

Our response: We have rewritten this paragraph (L112-120) to make it clearer. We only used two plasmids and it is enough for tagatose production. Plasmid pRS42K pTDH3-XYL1-tTDH3 is used for XR expression and plasmid pRS42H pTDH3-GDH-tCYC1 is used for GDH expression.

7. The concepts between 'equilibrium ratio' and 'ratio' were not clear. Authors claimed that the ratio of galactose and tagatose is around 7:3. The ratio of galactose and tagatose is 7:3 at 30 °C and around 5:5 at 60 °C. However, the production of tagatose by L-arabinose isomerase has been almost performed at 60-65 °C.
Our response: We compared the ratio at the same temperature (30 °C), which will be a fair comparison.

8. The many content of 'Introduction' was the same as that of 'Discussion'. Please remove the repeated contents.
Our response: We modified the introduction and discussion to remove the repeating parts.

9. Please, check 'Guide to authors'. For example, an abstract contains approximately 150 words according to the guide. However, this manuscript contained approximately 250 words. Reduce the words in the abstract. Moreover, the abstract should be intensively revised because of inaccurate expression. The primer design in Table I is not important. Transfer it to Supplementary data.
Our response: We modified the abstract (146 words). We have moved Table 1 to Supplementary Information (Table S1).

9. The citation is limited. I suggest the citation of more diverse and exact References.
Our response: We cited more references. These are list of papers newly cited:

1. Shin, K. C., Sim, D. H., Seo, M. J. & Oh, D. K. Increased Production of Food-Grade d-Tagatose from d-Galactose by Permeabilized and Immobilized Cells of Corynebacterium glutamicum, a GRAS Host, Expressing d-Galactose Isomerase from Geobacillus thermodenitrificans. J Agric Food Chem 64, 8146-8153, doi:10.1021/acs.jafc.6b03588 (2016).
2. Jayamuthunagai, J., Gautam, P., Srisowmeya, G. & Chakravarthy, M. Biocatalytic production of D-tagatose: A potential rare sugar with versatile applications. Crit Rev Food Sci Nutr 57, 3430-3437, doi:10.1080/10408398.2015.1126550 (2017).
3. Lim, B. C., Kim, H. J. & Oh, D. K. High production of D-tagatose by the addition of boric acid. Biotechnol Prog 23, 824-828, doi:10.1021/bp070056y (2007).
4. Park, C. S. et al. D-Allulose Production from D-Fructose by Permeabilized Recombinant Cells of Corynebacterium glutamicum Cells Expressing D-Allulose 3-Epimerase Flavonifractor plautii. PLoS One 11, e0160044, doi:10.1371/journal.pone.0160044 (2016).
5. Zhang, W., Yu, S., Zhang, T., Jiang, B. & Mu, W. Recent advances in d-allulose: Physiological functionalities, applications, and biological production. Trends in Food Science & Technology 54, 127-137, doi:https://doi.org/10.1016/j.tifs.2016.06.004 (2016).
6. Wei, N., Xu, H., Kim, S. R. & Jin, Y. S. Deletion of FPS1, encoding aquaglyceroporin Fps1p, improves xylose fermentation by engineered Saccharomyces cerevisiae. Appl Environ Microbiol 79, 3193-3201, doi:10.1128/AEM.00490-13 (2013).

7. Kwak, S. et al. Enhanced isoprenoid production from xylose by engineered Saccharomyces cerevisiae. Biotechnol Bioeng 114, 2581-2591, doi:10.1002/bit.26369 (2017).

Moreover, in Reference section, there were too many spelling errors and format errors. References should be revised point by point.
Our response: Thank you. We fixed them.

1) All journal names of references should be revised. For examples, the journal name of 'J Agric Food Chem' should be revised to 'J. Agric. Food Chem.'. The journal name of 'Nutrition and Food Sciences Research' should be revised to 'Nutr. Food Sci. Res.'.
2) Reference numbers 3-6, 11, 14-17, 19, 21-26, and 29: The names of microorganisms should be italicized.
3) Reference numbers 3-6, 10-13, 15, 16, 21-23, 25, and 27: The words of 'L-', 'D-',' 'l-', and 'd-' should be revised to the small capital 'L-', and 'D-'.
4) Reference numbers 2, 17, and 30: Title should be small letters except for the first word.
5) Reference 1: What is the meaning for 'US$ 183.50 (hardback)'.

Specific comments

1. Abstract: Please, include the lactose-related contents because the key word is 'lactose'
Our response: We added lactose related contents as suggested by the reviewer.

2. Abstract, L28, L41: The ratio of '3:7' is not exact value.
Our response: We specified a particular temperature (at 30 °C) where the ratio of 3:7 is observed.

3. Abstract, L31: Authors described that the oxidoreductases require cofactors. Thus, provide the solution for the requirement of cofactors.
Our response: NADPH for XR can be generated by oxidative pentose phosphate pathway and NAD^+ for GDH can be generated by TCA cycle from glucose metabolism.

4. Abstract, L38-39: How do you perform that "the expression levels of XR and GDH were adjusted to optimize tagatose production"
Our response: We constructed engineered yeast strains with different copy numbers of XR and GDH to evaluate the effect of expression levels of key enzymes on tagatose production.

5. Abstract, L40: Please, insert the term of 'in the reaction broth' after 'with a ratio of 9:1'.
Our response: We have added "in the reaction broth" after "with a ratio of 9:1" as suggested by the reviewer (Line 32).

Engineered yeast produced 37.7 g/L of tagatose from not galactose but lactose. The ratio of '9:1' is not exact value because the reaction broth contained lactose, ethanol, and galactitol. The ratio should be tagatose per galactose, lactose, ethanol, and galactitol.
Our response: Although the fermentation broth contained lactose, galactitol, the ratio of

tagatose and galactose in the fermentation broth is 9:1. Concentrations of lactose and galactitol were negligible. Please refer to Fig. S6.

6. Abstract, L46: Remove the word of 'L-arabinose isomerase' and insert the word of 'galactose'.
Our response: We removed 'L-arabinose isomerase' and inserted the word of 'galactose' as suggested by the reviewer (Line 36).

7. Introduction, L76-78: The explanation of the sentence is not correct because tagatose production was performed by not enzyme but immobilized cells.
Our response: We modified the sentence based on the reviewer's comment.

8. Introduction, L84: The ratio of '7:3' is not exact value. I suggest '5:5'.
Our response: The ratio is 7:3 at 30 °C so that we specified the temperature.

9. Introduction, L86: The word of 'S. cerevisiae' should be revised to 'Saccharomyces cerevisiae'.
Our response: We changed '*S. cerevisiae*' into '*Saccharomyces cerevisiae*' as suggested (Line 78).

10. Results, L104: The HPLC results of Fig. 1 should be provided in Supplementary data.
Our response: We included the HPLC chromatograms which were used for generating Fig. 1 as Fig. S1 in Supplementary Information.

11. Results, L116-119: NADPH-linked XR was coupled with NAD+-linked GDH but not NADP+-linked GDH. Explain the regeneration of cofactors.
Our response: XR and GDH coupled reaction can recycle cofactors. While XR prefers NADPH, NADH can be also used by XR. Produced NAD+ by XR can be used GDH. Also, additional NAD+ can be generated from TCA cycle and electron transport chain under aerobic conditions.

12. Results, L118-119: The HPLC results of Fig. 2 should be provided in Supplementary data.
Our response: We included the HPLC chromatograms used for generating Fig. 2 in Supplementary data as Fig. S2.

13. Results, L123: The HPLC results of Fig. 3 should be provided in Supplementary data.
Our response: We included the HPLC chromatogram used for generating Fig. 3 in Supplementary data as Fig. S4.

14. Results, L136-137: Please, explain more detail for EJ2g_XG_X, EJ2g_XG_G, and EJ2g_XG_XG.
Our response: We renamed the strains systematically. The changes are summarized in the following table. X and G denote XR and GDH and supercripts i and p denote integrated and plasmid copies, respectively.

Strains	Previous name	New name
1	EJ2g X	EJ2g_iX

2	EJ2g G	EJ2g_iG
3	EJ2g XG	EJ2g_iXiG
4	EJ2g_XG_X	EJ2g_iXiG_pX
5	EJ2g_XG_G	EJ2g_iXiG_pG
6	EJ2g_XG_XG	EJ2g_iXiG_pXpG

15. Results, L145: The difference between the yield of tagatose from lactose by EJ2g_XG_XG and the theoretical maximum yield should be described.
Our response: The tagatose yield from lactose was 0.33 g tagatose/g lactose which is equivalent to 62.7% of a theoretical maximum (0.526 g tagatose/g lactose).

L319-322, "The maxium theoretical tagatose yield (0.526 g tagatose/g lactose) by engineered yeast was calculated based on the molecular weights of tagatose (180.16 g/mol) and lactose (342.3 g/mol) with an assumption that glucose is not coverted into tagatose."

16. Results, L150-152: Please, described the total amount of lactose (g/L) at the fed-batch fermentation.
Our response: We added the total amount (114.21 g/L) of fed lactose in the revised manuscript.

Line 149-150, "After the fed-batch fermentation, 114.21 g/L of lactose was consumed and the titers of tagatose, galactose, and galactitol were 37.69 g/L, 4.41 g/L, and 8.46 g/L respectively."

17. Results, L157: The HPLC results of Fig. 6 at several fermentation times should be provided in Supplementary data.
Our response: We added the HPLC chromatograms used for generating Fig. 6 (Fig. 5 in the revised manuscript) in Supplementary Information as Fig. S6. We also added the HPLC chromatograph for Fig. 4 in Supplementary data as Fig. S5.

18. Results, L157: The ratio of '3:7' is not exact value. I suggest '5:5'.
Our response: We specified the temperature (30 °C) with the ratio of 7:3 to be accurate.

19. Results, L142: Please, explain the content of Fig. 5.
Our response: Fig. 5 is a summary of the tagatose yield of our engineered yeast strains as compared to that from isomerase reaction. We have deleted it in our revised manuscript since it seems redundant.

20. Results, 147-162: The experiments for tagatose production with increasing the total amount of lactose at the fed-batch fermentation should be performed.
Our response: If we increase lactose concentration, it will generate acetate and hence will inhibit tagatose production. That is why we used 50 g/L of lactose in the beginning and control the feeding lactose less than 20 g/L.

21. Results, 156: The ratio of '9:1' is not exact value because the reaction broth contained

lactose, ethanol, and galactitol. The ratio should be tagatose per galactose, lactose, ethanol, and galactitol.
Our response: Although the fermentation broth contained lactose, galactitol, the ratio of tagatose and galactose in the fermentation broth is 9:1. Concentrations of lactose and galactitol were negligible.

22. Discussion, 170-174: I suggest 'L-arabinose isomerase' instead of xylose isomerase.
Our response: Xylose isomerase (XI) vs. xylose reductase (XR) and xylitol dehydrogenase (XDH) for the conversion of xylose into xylulose have been extensively compared in the context of rate, yield, and redox blance in bacteria and yeast (fungi). In contrast, comparison of arabinose isomerase (AI) and arabinose reductase (AR) / arabitol dehydrogenase (ADH) have not been studied much yet. As our intention was to compare the isomerase and oxidoreductase pathways, we feel that the comparison of XI vs. XR/XDH is more appropriate than AI vs. AR/ADH.

23. Discussion, 178-182, 227-228: The explanation of the sentence is not correct because tagatose production was performed by not enzyme but immobilized cells. The separation process using enzyme reaction is simpler than that using fermentation.
Our response: We meant the separation of tagatose from a mixture of tagatose and galactose rather than the separation of the tagatose from immobilized enzyme. Separation of sugar isomers (tagatose and galactose) requires a relatively expensive process, such as simulated moving bed (SMB) charomatography.

24. Materials and Methods, 244: Describe yeast strain.
Our response: We described yeast strains in detail in our revised manuscript (Table 1).

25. Materials and Methods, 246: The word of 'Ampicillin' should be revised to 'ampicillin'.
Our response: We corrected it.

26. Materials and Methods, 244: Cell mass should determined using a calibration curve derived from the correlation between the dry cell weight and the OD600 of yeast fermentation broth.
Our response: According to our cell mass determination experiments, the conversion factor was 0.454 g/L * OD600. We added this into Materials and Methods (Line 310).

27. Tables: Please, transfer Table I and Table II to Supplementary data.
Our response: We moved Table I and Table II to Supplementary Information (Table S1 and Table S3) as suggested.

28. Figures:
1) In the all figures, fermentation conditions and method should be included.
Our response: We have added fermentation conditions and method in our revised manuscript
2) Combine Fig. 1 and Fig. 2.
Our response: We think it is better to leave it as separate version as EJ2g with empty plasmid p42k was used as control for Fig. 2.
3) Please, transfer Fig. 5 to Supplementary data because the explanation in the text is enough.
Our response: We deleted Fig. 5 as it contained the same results of Fig. 4.
4) The term of '~30%' in Fig. 5 and its legend should be revised to '~50%'.

Our response: We deleted Fig. 5.
5) The references in Fig. 5 should be changed to those showing '~50%'.
Our response: We deleted Fig. 5
6) The OD600 in Fig. 6B should be revised to dry cell weight (g/L).
Our response: we have converted OD600 to DCW (g/L).

References cited

Kim, S. R., S. J. Ha, Kong, II and Y. S. Jin (2012). "High expression of XYL2 coding for xylitol dehydrogenase is necessary for efficient xylose fermentation by engineered Saccharomyces cerevisiae." Metab Eng **14**(4): 336-343.
Kim, S. R., Y. C. Park, Y. S. Jin and J. H. Seo (2013). "Strain engineering of Saccharomyces cerevisiae for enhanced xylose metabolism." Biotechnol Adv **31**(6): 851-861.
Kim, S. R., J. M. Skerker, W. Kang, A. Lesmana, N. Wei, A. P. Arkin and Y. S. Jin (2013). "Rational and evolutionary engineering approaches uncover a small set of genetic changes efficient for rapid xylose fermentation in Saccharomyces cerevisiae." PLoS One **8**(2): e57048.
Kwak, S., S. R. Kim, H. Xu, G. C. Zhang, S. Lane, H. Kim and Y. S. Jin (2017). "Enhanced isoprenoid production from xylose by engineered Saccharomyces cerevisiae." Biotechnol Bioeng **114**(11): 2581-2591.
Oh, E. J., S. J. Ha, S. Rin Kim, W. H. Lee, J. M. Galazka, J. H. Cate and Y. S. Jin (2013). "Enhanced xylitol production through simultaneous co-utilization of cellobiose and xylose by engineered Saccharomyces cerevisiae." Metab Eng **15**: 226-234.

Reviewers' comments:

Reviewer #1 (Remarks to the Author):

The revised manuscript is much improved, and addresses all the concerns raised by Reviewers 1 and 2 satisfactorily. Thus publication in Nature Communications is recommended.

Reviewer #2 (Remarks to the Author):

I find that the authors have convincingly addressed all questions, comments, and concerns regarding the initial draft, and I believe the revised manuscript is ready for publication. I have no further issues nor comments.

Reviewer #3 (Remarks to the Author):

The response of authors to my comments was carefully checked. Some contents were resolved. However, some critical problems are still remained. Moreover, some authors' responses are not enough. Thus, this second version manuscript should be revised.

Critical problems:
1. Although the equilibrium ratio of galactose and tagatose was emphasized, the conversion ratio of tagatose was skipped.
Authors described the response for specific comment 16 of "After the fed-batch fermentation, 114.21 g/L of lactose was consumed and the titers of tagatose, galactose, and galactitol were 37.69 g/L, 4.41 g/L, and 8.46 g/L respectively." The data indicate that the conversion ratios of tagatose, galactose, and galactitol were 74.5%, 8.7%, and 16.7%, respectively. Thus, the response for specific comment 5 of "Concentrations of lactose and galactitol were negligible" was not correct.
In enzymatic biotransformation, there are only galactose and tagatose. However, in the fermentation in this study, fermentation broth contained not only galactose and tagatose but also 16.7% galactitol. Thus, the conversion ratio of tagatose, 74.5%, should be included and emphasized in the second revised manuscript.

2. The comparison of your system and previously reported enzymatic biotransformation was not clear.
Engineered yeast produced 37.7 g/L tagatose from lactose for 300 h with a productivity of 0.126 g/L/h. L-Arabinose isomerase converted 300 g/L galactose into 158 g/L tagatose for 20 h in the absence of boric acid, with a yield of 52.7% and a productivity of 7.9 g/L/h. (Reference, Lim, B. C. et al. Biotechnol. Prog. 23, 824-828 (2007).). I think that galactose at higher concentration was produced from lactose at higher concentration with higher productivity by beta-galactosidase, comparing with those of tagatose production from galactose. Thus, I think that enzymatic biotransformation shows higher productivity and concentration of tagatose than those of your fermentation results. Authors should be cited on the reference of lactose hydrolysis and discuss the productivities and final concentrations of tagatose from lactose for biotransformation and fermentation. The following references about the biotransformation of lactose into tagatose were also considered, compared, and discussed.
1) Jayamuthunagai, J. et. al. Bioresour. Technol. 235, 250-255 (2017).
2) Zhan, Y. et. al. J. Agric. Food Chem. 62(11), 2412-2417 (2014).
3) Wanarska, M. and Kur. J. Microb Cell Fact. 11, 113 (2012).
This equilibrium of galactose and tagatose was 47.3%: 52.7% at 60 °C. However, authors claimed only that the equilibrium of galactose and tagatose was 70%: 30% at 30 °C. The commercial production of tagatose has been performed at 60 °C. Therefore, the detailed description on the equilibrium of galactose and tagatose by varying temperature should be required.

The industrial production of tagatose has been performed from lactose by two-step reaction with beta-galctosidase and L-arabinose isomerase. Galactose was easily obtained from lactose by beta-galctosidase. Thus, your response for general comment 3 was not exact.

3. The amounts of gactactitol in this manuscript were not exact.
For specific comment 16 of "After the fed-batch fermentation, 114.21 g/L of lactose was consumed and the titers of tagatose, galactose, and galactitol were 37.69 g/L, 4.41 g/L, and 8.46 g/L respectively.". The response for specific comment 5 of "Concentrations of galactitol were negligible.". This is confusing.

4. The optimization data for tagatose production in a bioreactor were not provided.
Your response 20 of "That is why we used 50 g/L of lactose in the beginning and control the feeding lactose less than 20 g/L." should be provided by the data. Please show acetate accumulation and tagatose inhibition via the data on the fermentation above 50 g/L of lactose and fermentation at the feeding lactose more than 20 g/L.
Authors described the response for specific comment 16 of "After the fed-batch fermentation, 114.21 g/L of lactose was consumed." Please, show the results about the total amount of lactose (g/L) at the fed-batch fermentation above 114.21 g/L. For an example, when the total amount of lactose (g/L) at the fed-batch fermentation is 150 g/L, show the increase or decrease of tagatose production.

5. Is the equilibrium ratio of galactose and tagatose 1:9? The result should be verified by the reverse reaction of tagatose into galactose. I think that it is not equilibrium ratio but conversion ratio. This should be clearly defined.
For the determination of the exact ratio, whole cell reaction using the harvested cells of engineered yeast should be performed with galactose, not lactose, to produce tagatose. Moreover, whole cell reaction should be also performed with tagatose to produce galactose.

6. I do not understand the HPLC data (Figs. S1, S2, S4, and S6).
Please provide exact quantitative data (or ratios) for all substrates and products, including lactose, galactose, tagatose, and galactitol.

<u>Responses to Reviewers' Comments</u>

Reviewer comments are copied in black while author responses have been written in blue. The changes to the manuscript are shown in red font.

Reviewers' comments:

Reviewer #1 (Remarks to the Author):

The revised manuscript is much improved, and addresses all the concerns raised by Reviewers 1 and 2 satisfactorily. Thus publication in Nature Communications is recommended.
<u>Response:</u> Thank you.

Reviewer #2 (Remarks to the Author):

I find that the authors have convincingly addressed all questions, comments, and concerns regarding the initial draft, and I believe the revised manuscript is ready for publication. I have no further issues nor comments.

Michael Naesby
<u>Response:</u> Thank you Dr. Naesby.

Reviewer #3 (Remarks to the Author):

The response of authors to my comments was carefully checked. Some contents were resolved. However, some critical problems are still remained. Moreover, some authors' responses are not enough. Thus, this second version manuscript should be revised.
<u>Response:</u> We appreciate the comments by Reviewer #3 and revised our manuscript to address the critical problems as follow.

Critical problems:
1. Although the equilibrium ratio of galactose and tagatose was emphasized, the conversion ratio of tagatose was skipped.
Authors described the response for specific comment 16 of "After the fed-batch fermentation, 114.21 g/L of lactose was consumed and the titers of tagatose, galactose, and galactitol were 37.69 g/L, 4.41 g/L, and 8.46 g/L respectively." The data indicate that the conversion ratios of tagatose, galactose, and galactitol were 74.5%, 8.7%, and 16.7%, respectively. Thus, the response for specific comment 5 of "Concentrations of lactose and galactitol were negligible" was not correct.
In enzymatic biotransformation, there are only galactose and tagatose. However, in the fermentation in this study, fermentation broth contained not only galactose and tagatose but also 16.7% galactitol. Thus, the conversion ratio of tagatose, 74.5%, should be included and emphasized in the second revised manuscript.
<u>Response:</u> As suggested, we added the conversion ratios of lactose into tagatose, galactose, and galactitol into main text (**Line 152-153**). We agree that "Concentrations

of lactose and galactitol were negligible" was not correct. We intended to mean "Concentrations of ethanol, acetate, and glycerol" in the response. Sorry for the mistake.

Line 152-153, As such, the conversion ratios of tagatose, galactose, and galactitol were 74.5%, 8.7%, and 16.7%, respectively.

2. The comparison of your system and previously reported enzymatic biotransformation was not clear.
Engineered yeast produced 37.7 g/L tagatose from lactose for 300 h with a productivity of 0.126 g/L/h. L-Arabinose isomerase converted 300 g/L galactose into 158 g/L tagatose for 20 h in the absence of boric acid, with a yield of 52.7% and a productivity of 7.9 g/L/h. (Reference, Lim, B. C. et al. Biotechnol. Prog. 23, 824-828 (2007).). I think that galactose at higher concentration was produced from lactose at higher concentration with higher productivity by beta-galactosidase, comparing with those of tagatose production from galactose. Thus, I think that enzymatic biotransformation shows higher productivity and concentration of tagatose than those of your fermentation results.
Response: We agree that reported enzyme-based biotransformation showed much higher productivities than our productivity. However, the conversion yields (53%) were lower than our yield (74.5%). The main purpose of our report is to show the feasibility of replacing an *in vitro* isomerase reaction which inevitably results in a mixture of substrate and product with an unfavorable ratio with a corresponding pair of oxido-reductase enzymes *in vivo* to increase the conversion ratio. In order to provide a fair comparison of fermentation and enzyme-based tagatose production from lactose and galactose as suggested by Reviewer #3, we cited the reference (Lim et al.) and added the comparison in Discussion (**Line 209-217**).

Authors should be cited on the reference of lactose hydrolysis and discuss the productivities and final concentrations of tagatose from lactose for biotransformation and fermentation. The following references about the biotransformation of lactose into tagatose were also considered, compared, and discussed.
1) Jayamuthunagai, J. et. al. Bioresour. Technol. 235, 250-255 (2017).
2) Zhan, Y. et. al. J. Agric. Food Chem. 62(11), 2412-2417 (2014).
3) Wanarska, M. and Kur. J. Microb Cell Fact. 11, 113 (2012).
Response: Thanks for providing the references. As Jayamuthunagai et al. conducted sequential enzymatic reactions of beta-galactosidase and arabinose isomerase, their productivity is not comparable with our result. However, Zhan et al. and Wanarska et al. performed simultaneous hydrolysis and isomerization of lactose which is comparable with our study. Therefore, we cited their productivities and titers of tagatose (**Line 209-217**).

Line 209-217, In addition to galactitol accumulation as a byproduct, the productivity and titer of tagatose from lactose by our engineered yeast need to be further improved. While the productivity (0.126 g/L·h) and titer (37.69 g/L) of tagatose production from lactose by our engineered yeast are comparable with the reported productivities (0.103-0.896 g/L·h) and titers (14.8-21.5 g/L) by enzyme-based tagatose production from

lactose [18,33], they are much lower than the productivity (7.9 g /L·h) and titer (158 g/L) by enzyme-based tagatose production from galactose [5]. Continuous fermentation with cell-recycling might be a possible approach to improve the productivity of tagatose production from lactose by our engineered yeast.

This equilibrium of galactose and tagatose was 47.3%: 52.7% at 60 °C. However, authors claimed only that the equilibrium of galactose and tagatose was 70%: 30% at 30 °C. The commercial production of tagatose has been performed at 60 °C. Therefore, the detailed description on the equilibrium of galactose and tagatose by varying temperature should be required.
Response: We clarified temperature dependence of the equilibrium ratio of galactose and tagatose in main text (**Line 64-65**) as suggested.

Line 64-65, Although the ratio of galactose and tagatose after enzymatic isomerization can be shifted to 4:6 by increasing reaction temperatures to 60°C,

The industrial production of tagatose has been performed from lactose by two-step reaction with beta-galctosidase and L-arabinose isomerase. Galactose was easily obtained from lactose by beta-galctosidase. Thus, your response for general comment 3 was not exact.
Response: To the best of knowledge, commercial productions of tagatose have been conducted using lactose and beta-galactosidase but tagatose prices are not competitive to HFCS. We reason that current bottlenecks of rare sugar production are not in production but in overall process, including the purification. While lactose can be easily hydrolyzed into glucose and galactose using beta-galactosidase, the hydrolysis reaction will produce a mixture of glucose and galactose. As such, additional purification steps for separating galactose from the mixture will be necessary. In our fermentation process, lactose is hydrolyzed into glucose and galactose intracellularly. The intracellularly produced glucose is metabolized for growth and maintenance of engineered yeast and galactose is converted into tagatose, making it possible to bypass the galactose purification step.

3. The amounts of gactactitol in this manuscript were not exact.
For specific comment 16 of “After the fed-batch fermentation, 114.21 g/L of lactose was consumed and the titers of tagatose, galactose, and galactitol were 37.69 g/L, 4.41 g/L, and 8.46 g/L respectively.”. The response for specific comment 5 of “Concentrations of galactitol were negligible.”. This is confusing.
Response: The statement “Concentrations of lactose and galactitol were negligible” in our previous response to Specific comment 5 was incorrect. We meant to say “Concentrations of ethanol, acetate, and glycerol were negligible”. We regret the mistake. The statement in our response to Specific comment 16, “After the fed-batch fermentation, 114.21 g/L of lactose was consumed and the titers of tagatose, galactose, and galactitol were 37.69 g/L, 4.41 g/L, and 8.46 g/L respectively.” is correct.

4. The optimization data for tagatose production in a bioreactor were not provided.

Your response 20 of “That is why we used 50 g/L of lactose in the beginning and control the feeding lactose less than 20 g/L.” should be provided by the data. Please show acetate accumulation and tagatose inhibition via the data on the fermentation above 50 g/L of lactose and fermentation at the feeding lactose more than 20 g/L.
Response: In our earlier trials of fed-batch fermentation as shown below, we fed 54.31 g/L of lactose when yeast depleted initially added 49.59 g/L of lactose. We observed a substantial amount (4.68 g/L) of acetate accumulation and cells stopped using lactose due to the excessive acetate accumulation. We think acetate did not inhibit tagatose production via specific interactions with the enzymes involved in tagatose production but inhibited lactose uptake.

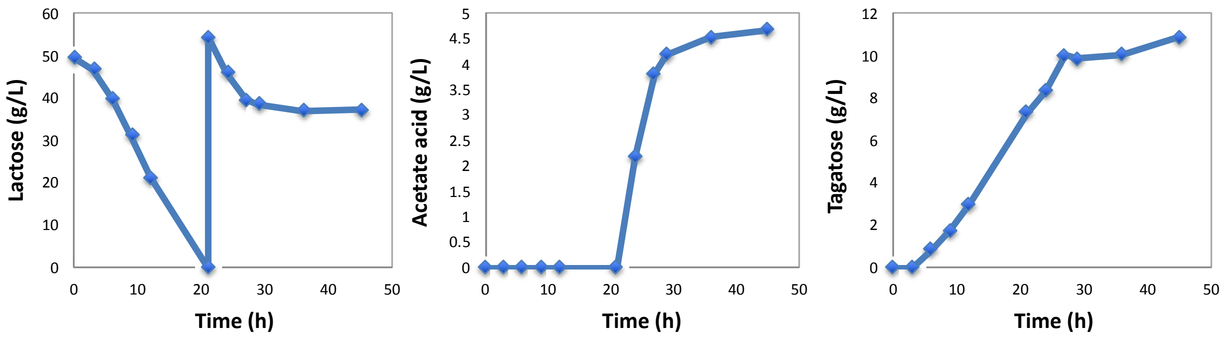

When we reduced the lactose concentration levels lower than 20 g/L, we did not observe the acetate accumulation anymore. Further studies to investigate the effects of lactose concentrations on physiological changes of our engineered yeast during fed-batch fermentation will be necessary.

Authors described the response for specific comment 16 of “After the fed-batch fermentation, 114.21 g/L of lactose was consumed.” Please, show the results about the total amount of lactose (g/L) at the fed-batch fermentation above 114.21 g/L. For an example, when the total amount of lactose (g/L) at the fed-batch fermentation is 150 g/L, show the increase or decrease of tagatose production.
Response: We also wonder whether or not tagatose production can be further increased during fed-batch fermentation. We presume that our system can achieve higher tagatose titer via extended fed-batch fermentation considering the continuing trend of tagatose production of strain EJ2g_iXiG_pXpG in Figure 5. Due to limitations of the laboratory fermentation environment in our study, including fermentor volume and medium composition, we plan to conduct further studies with an industrial partner.

5. Is the equilibrium ratio of galactose and tagatose 1:9? The result should be verified by the reverse reaction of tagatose into galactose. I think that it is not equilibrium ratio but conversion ratio. This should be clearly defined.
For the determination of the exact ratio, whole cell reaction using the harvested cells of engineered yeast should be performed with galactose, not lactose, to produce tagatose. Moreover, whole cell reaction should be also performed with tagatose to produce galactose.

Response: We had not mentioned the ratio of 1:9 as an equilibrium ratio of galactose and tagatose in our manuscript, but we agree that 1:9 is not an equilibrium ratio but a conversion ratio. We changed our text to clarify that (**L89**).

6. I do not understand the HPLC data (Figs. S1, S2, S4, and S6).
Please provide exact quantitative data (or ratios) for all substrates and products, including lactose, galactose, tagatose, and galactitol.
Response: We provided an Excel spreadsheet containing the concentrations of lactose, galactose, tagatose, and galactitol for Fig 1, 2, 3, 4, and 5 and noted in Data availability (**Line 336-337**).

Line 336-337, The source data underlying Figs 1, 2, 3, 4 and 5 are provided as a Source Data file.

REVIEWERS' COMMENTS:

Reviewer #3 (Remarks to the Author):

The revised manuscript is addressed for all my concerns. Now, the manuscript is acceptable at Nature Communications.
